# Supplementary material for: New Panx-1 Blockers: Synthesis, Biological Evaluation and Molecular Dynamic Studies
Source: Int J Mol Sci. 2022 Apr 27;23(9):4827. doi: 10.3390/ijms23094827 (PMC9103715; doi:10.3390/ijms23094827)
Supplement: Supplementary file 1 [file ijms-23-04827-s001.zip › ijms-1690208-supplementary.pdf]

Supporting Information

For

New Panx-1 blockers: synthesis, biological evaluation and molecular dynamic studies

Letizia Crocetti [1], Gabriella Guerrini [1], Maria Paola Giovannoni [1,], Fabrizio Melani [1], Silvia Lamanna [1], Lorenzo Di Cesare Mannelli [2], Elena Lucarini [2], Carla Ghelardini [2], Junjie Wang [3] and Gerhard Dahl [3]*

[1] Neurofarba, Pharmaceutical and Nutraceutical Section, University of Florence, Via Ugo Schiff 6, 50019, Sesto Fiorentino, Italy

[2] Neurofarba, Pharmacology and Toxicology Section, University of Florence, Viale Pieraccini 6, 50139 Florence, Italy;

[3] Department of Physiology and Biophysics University of Miami School of Medicine 1600 N.W. 10th Avenue Miami, Florida 33136

***Corresponding Author**

Prof. Maria Paola Giovannoni

Department of Neurofarba,

University of Florence, Via Ugo Schiff 6,

Sesto Fiorentino, Firenze 50019, Italy.

Tel. +39 055 4573682

Email: mariapaola.giovannoni@unifi.it

Table of Contents:

1. ^{1}H-NMR and ^{13}C-NMR spectra of final compounds **3b**, **3d**, **3e**, **7a-g**, **13**, **17**, **18c**, **19b**, **19c**, **24**, **28**, **30** and of some reaction intermediates (**15b**, **15c**, **16b**, **16c**, **25**).
2. Elemental analysis (**Table S1**).
3. Dose-response curves of compounds **3c**, **4** and **7d** (**Figures S1-S3**).

^{1}H-NMR and ^{13}C-NMR spectra of compound **3b**

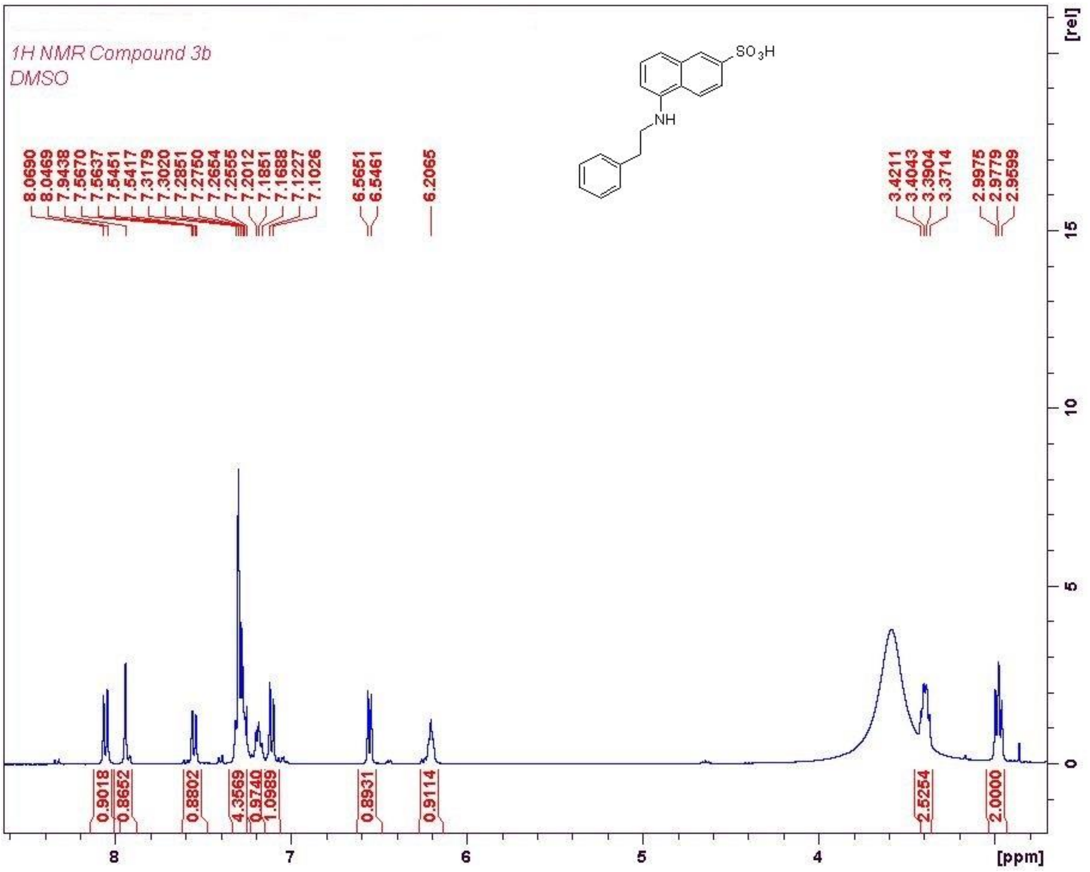

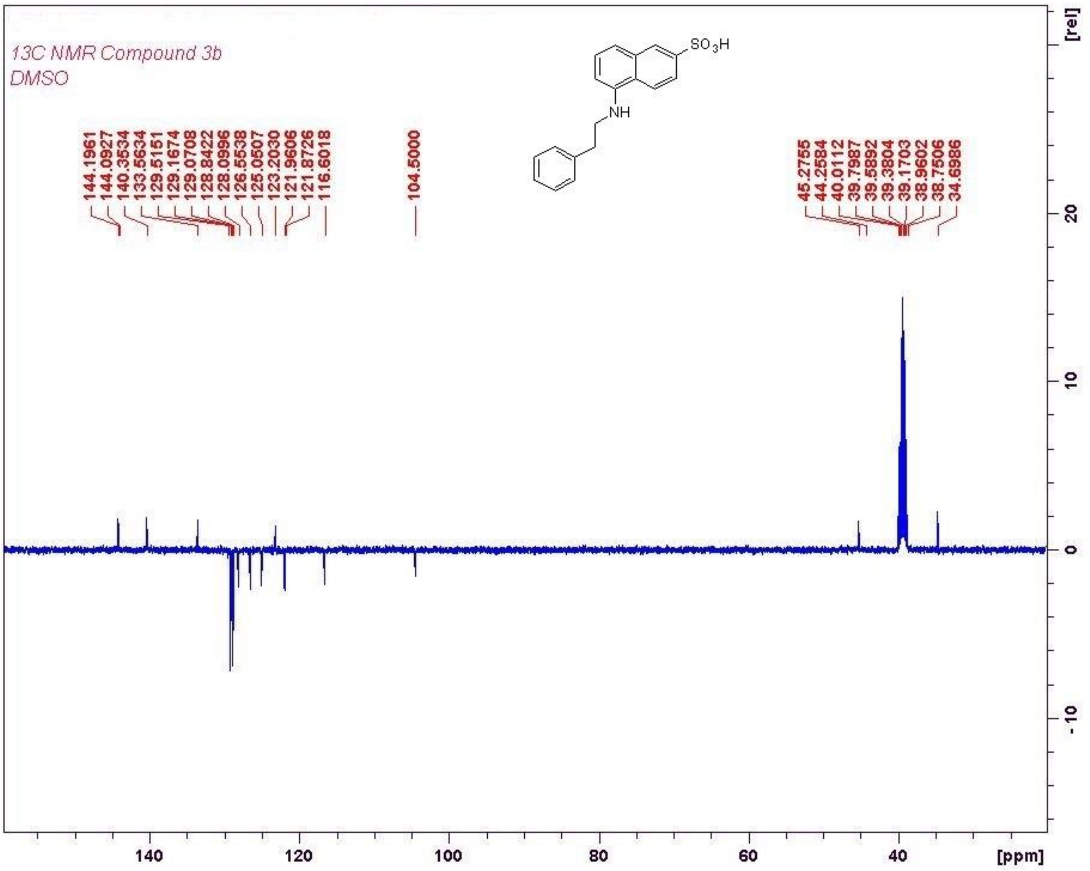

^{1}H-NMR and ^{13}C-NMR spectra of compound **3d**

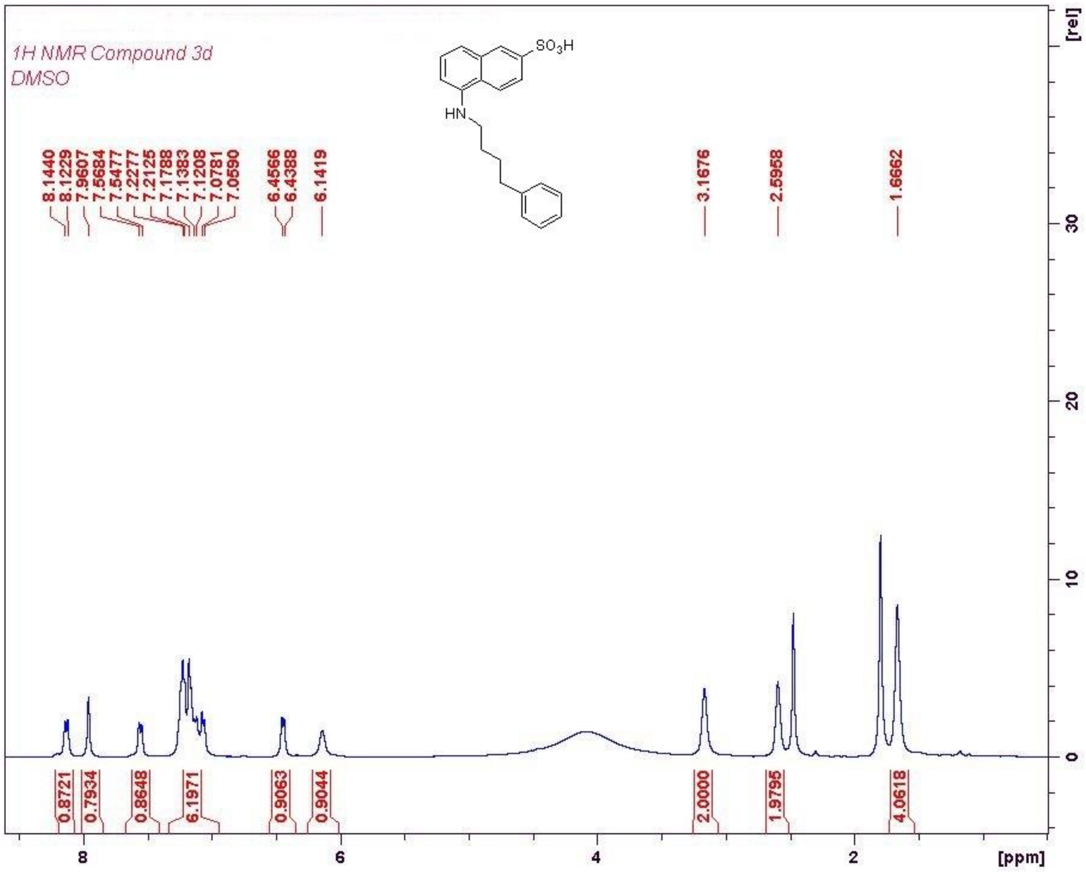

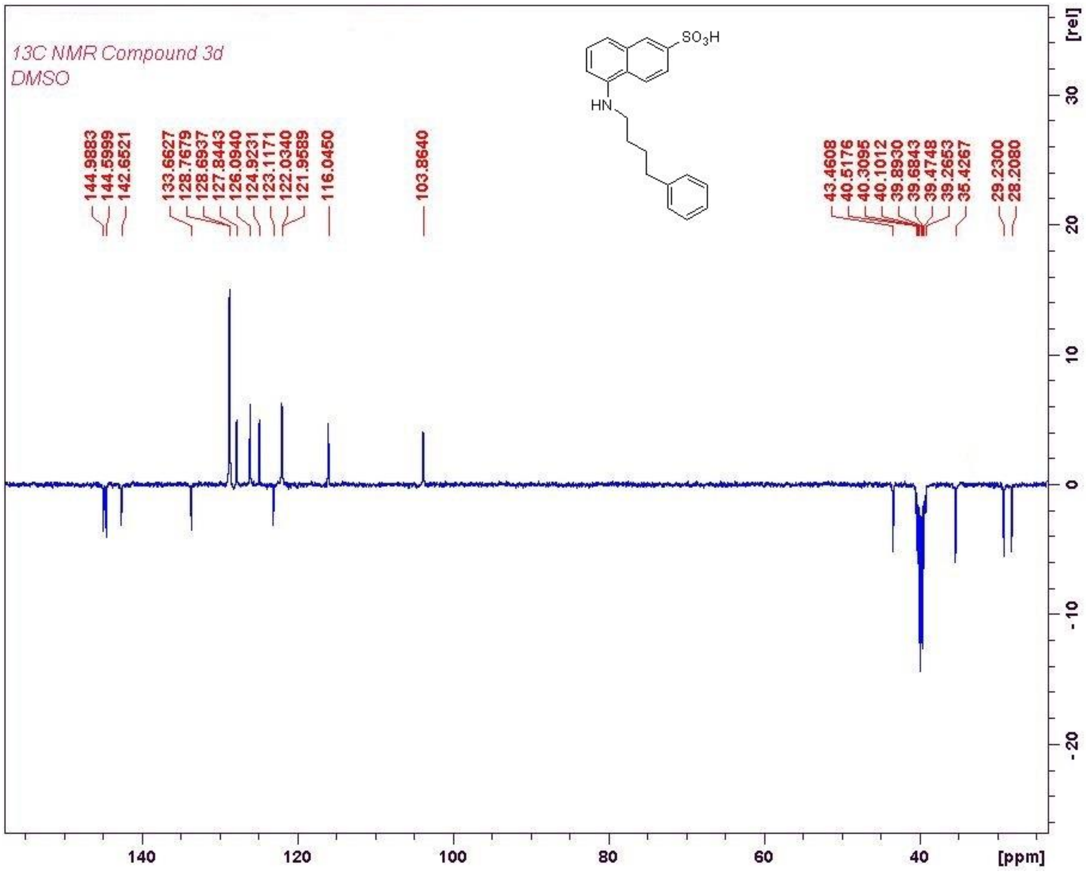

^{1}H-NMR and ^{13}C-NMR spectra of compound **3e**

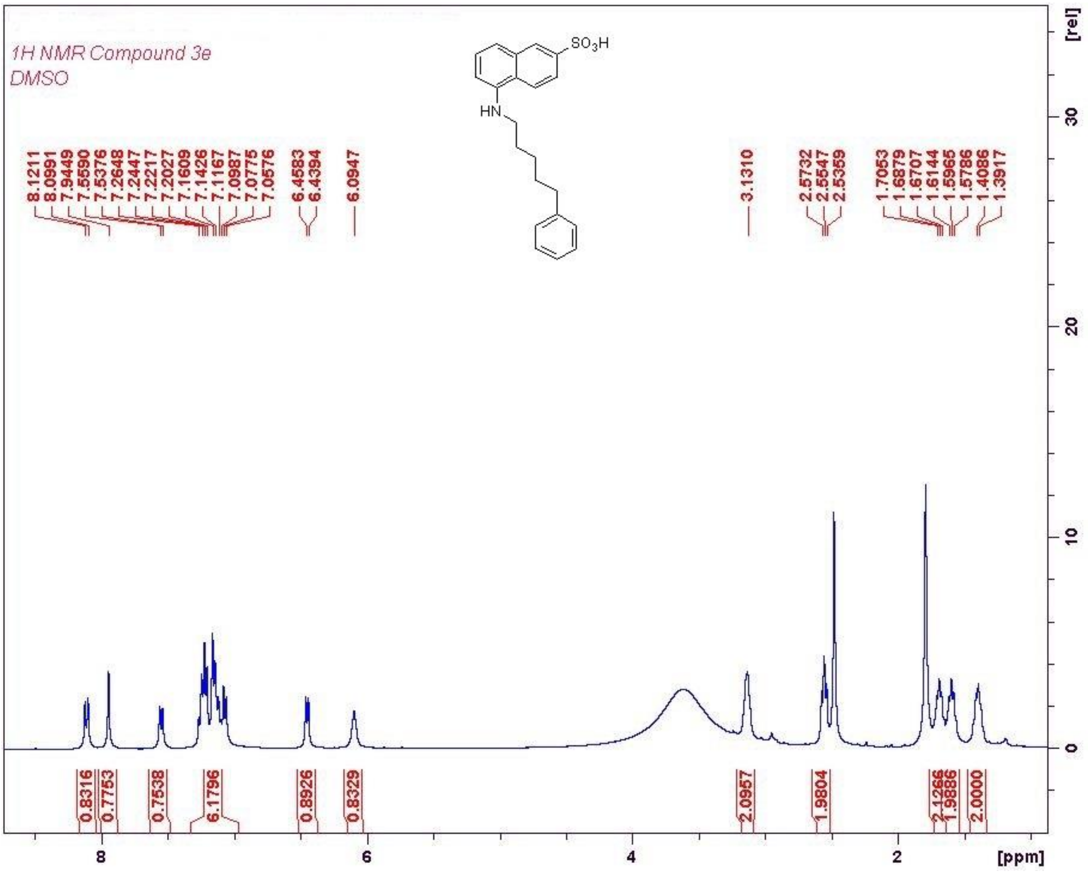

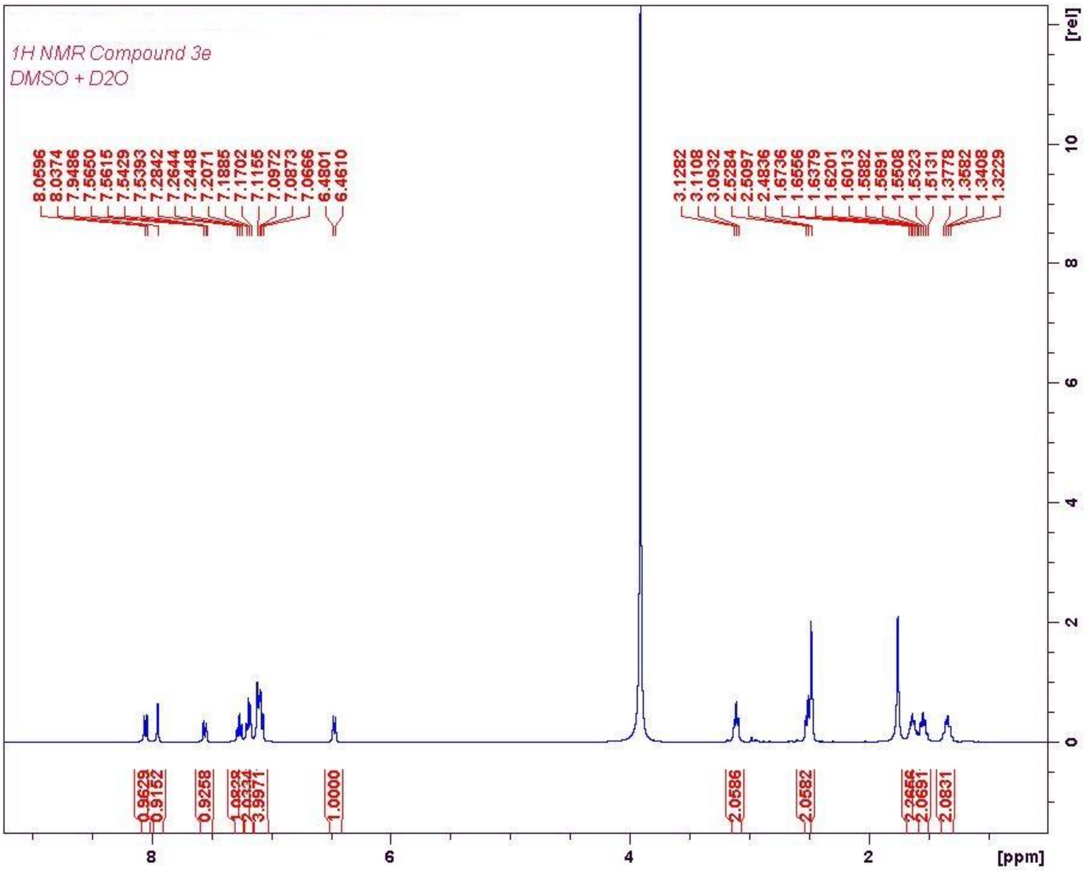

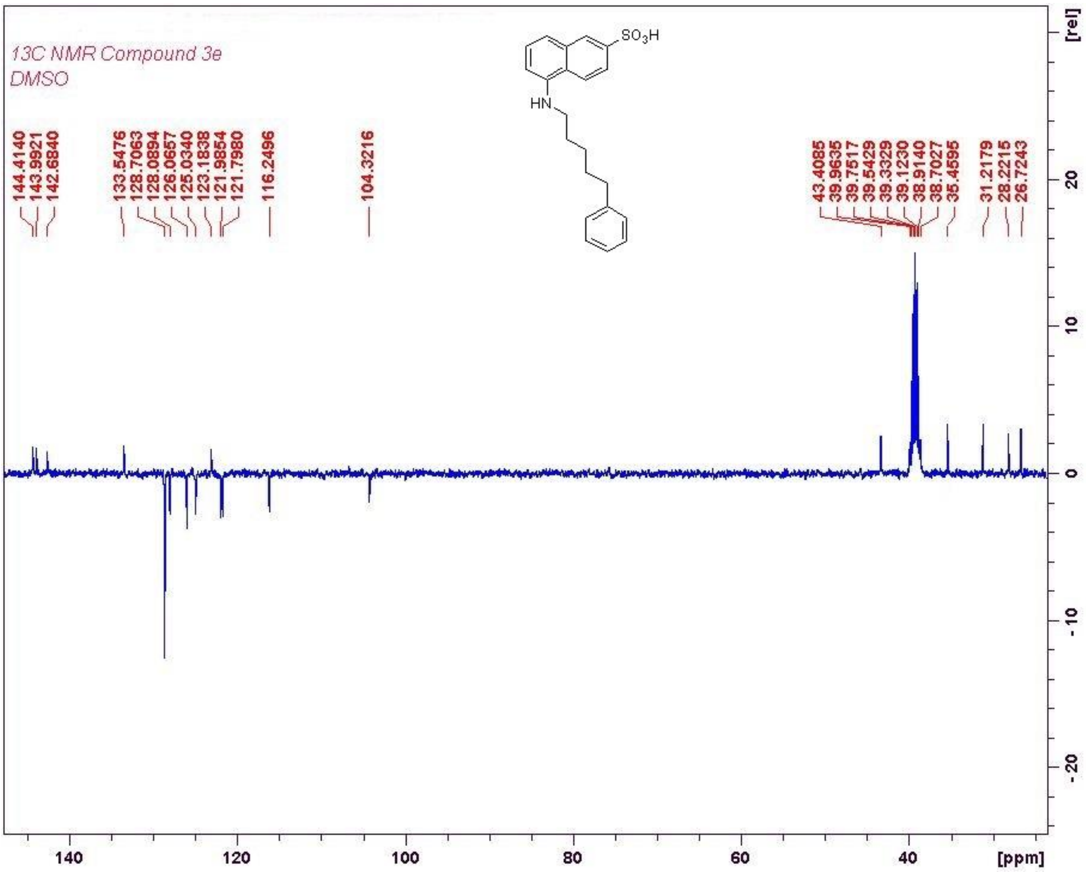

13C NMR Compound 3e
DMSO
SO_3H
HN
144.4140
143.9921
142.6840
133.5476
128.7063
128.0894
126.0657
125.0340
123.1838
121.9854
121.7980
116.2496
104.3216
43.4085
39.9635
39.7517
39.5429
39.3329
39.1230
38.9140
38.7027
35.4595
31.2179
28.2215
26.7243
[rel]
[ppm]

^{1}H-NMR and ^{13}C-NMR spectra of compound **7a**

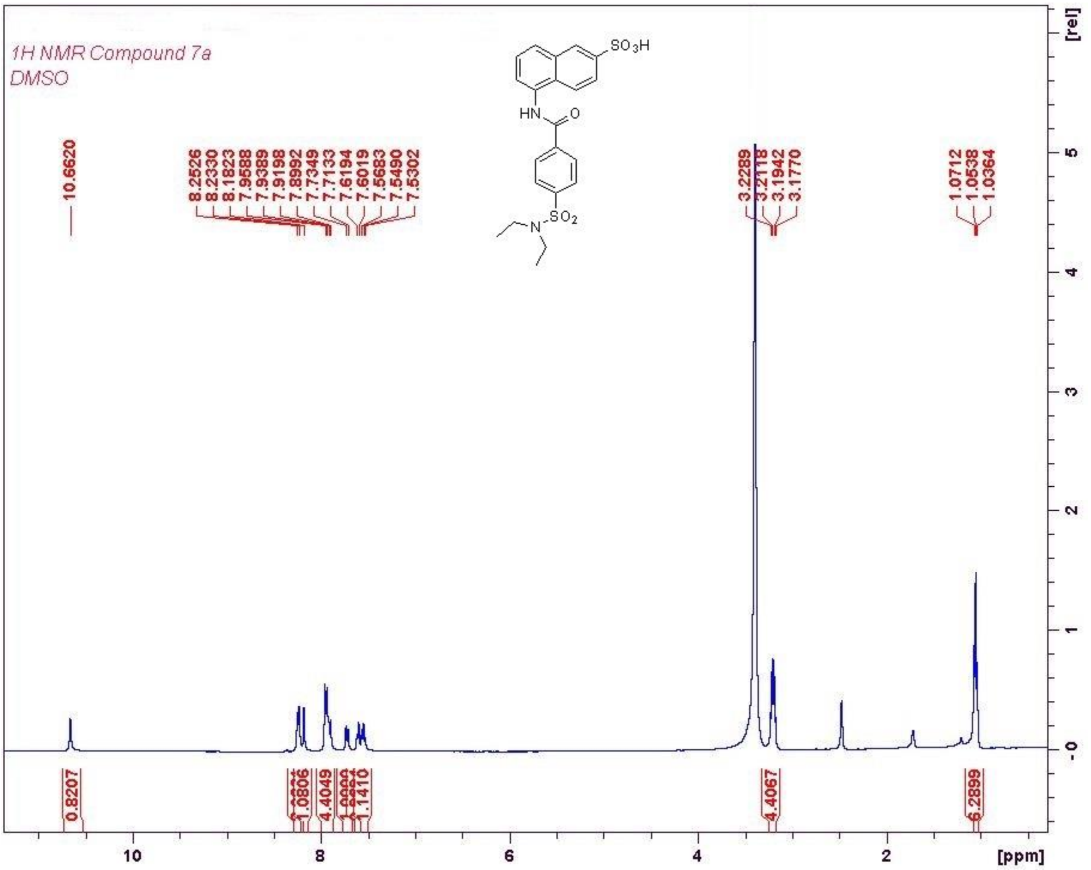

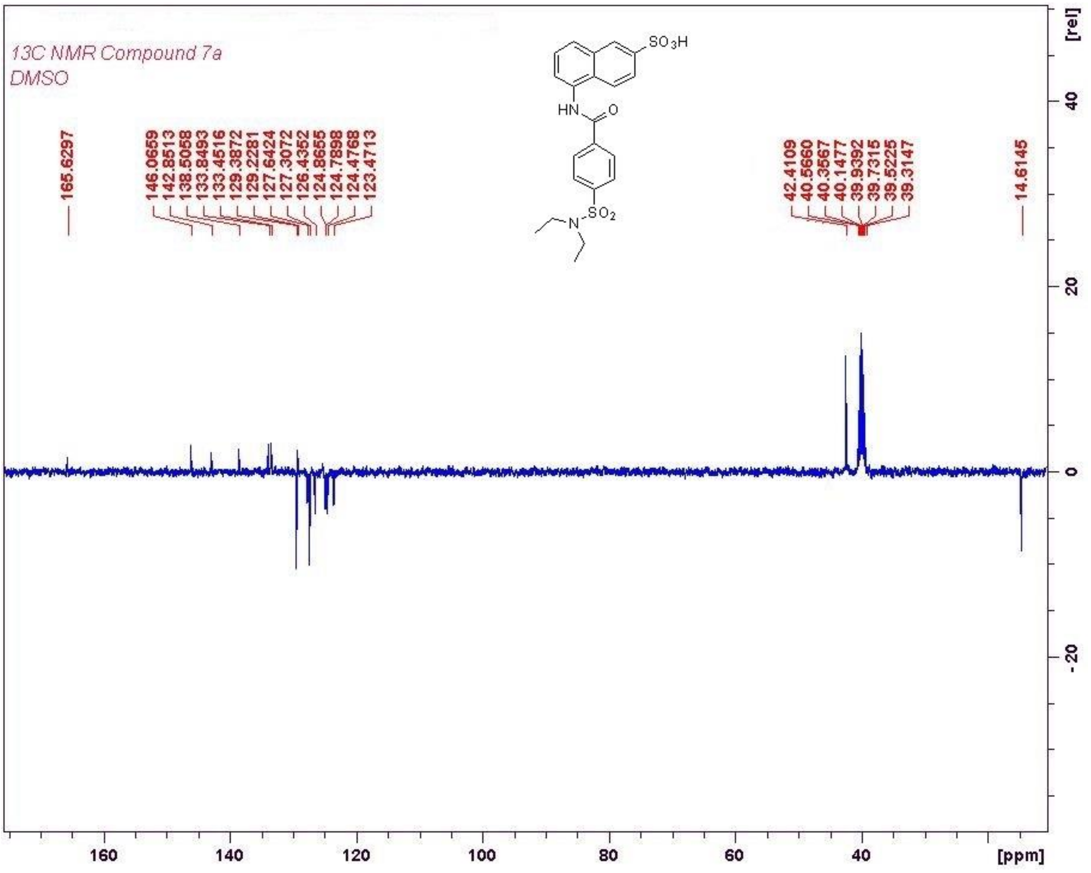

^{1}H-NMR and ^{13}C-NMR spectra of compound **7b**

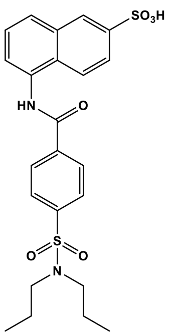

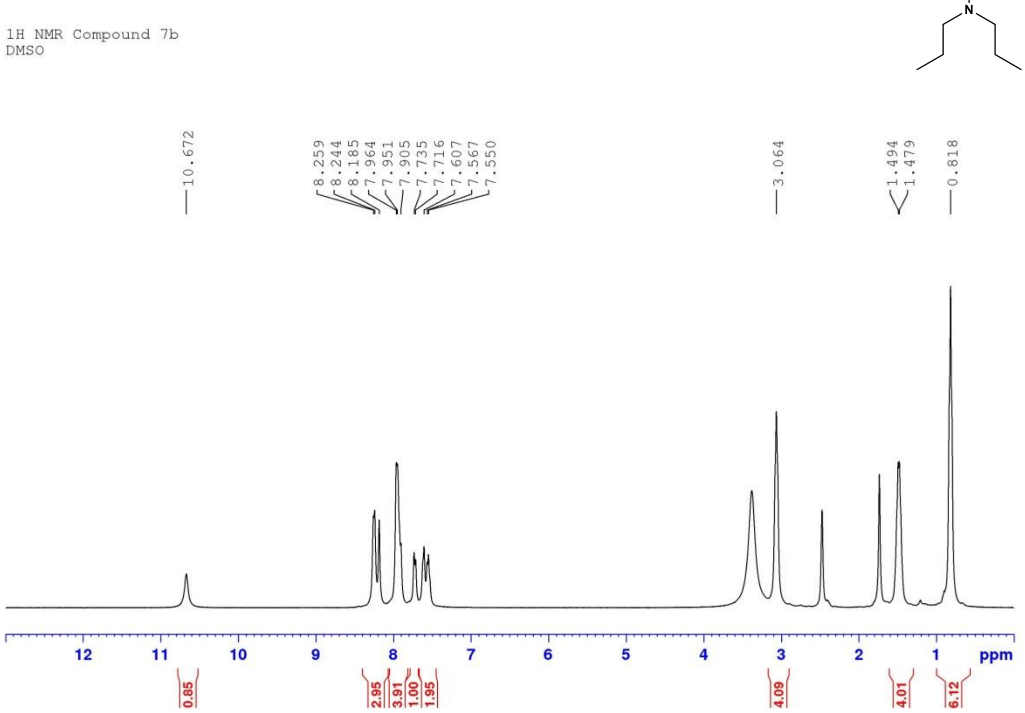

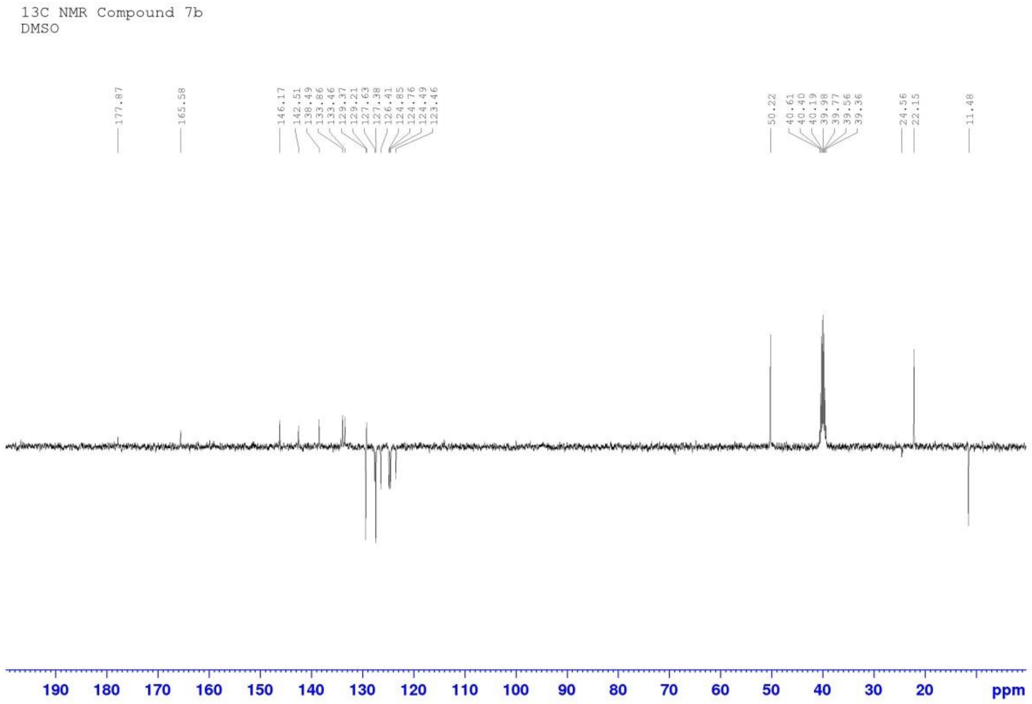

^{1}H-NMR and ^{13}C-NMR spectra of compound **7c**

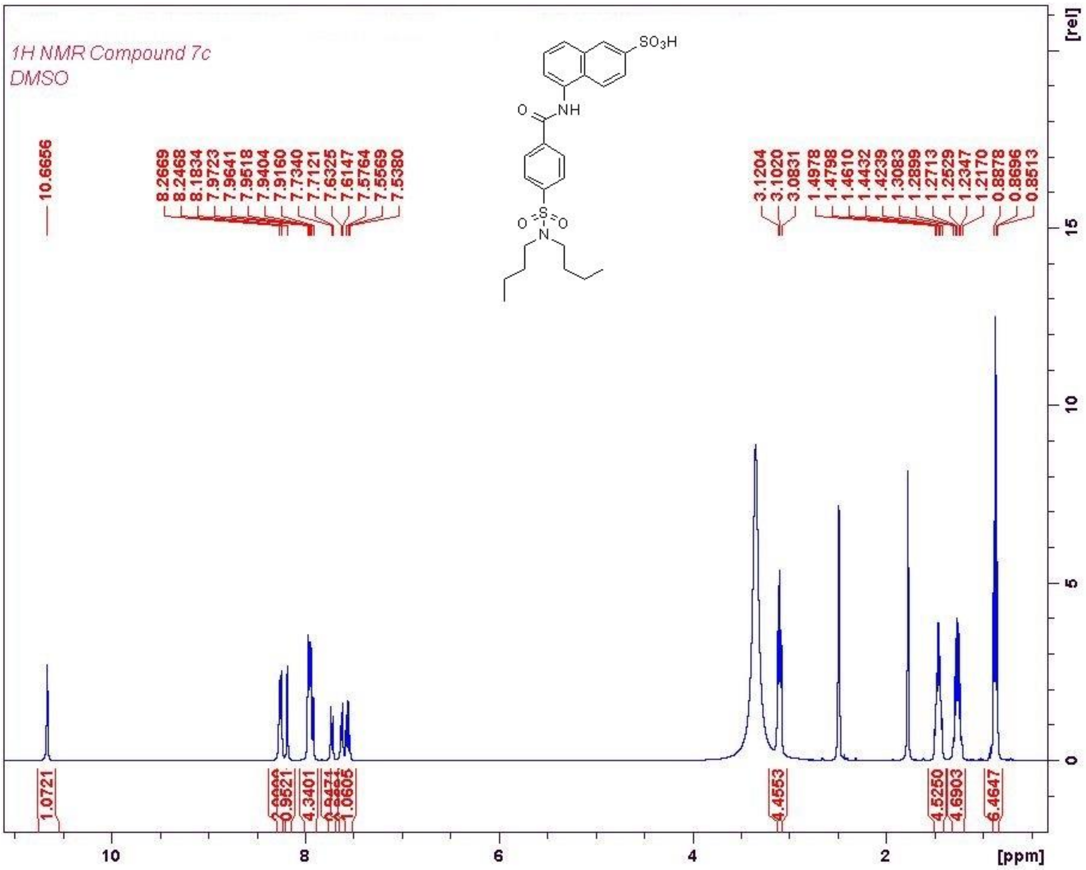

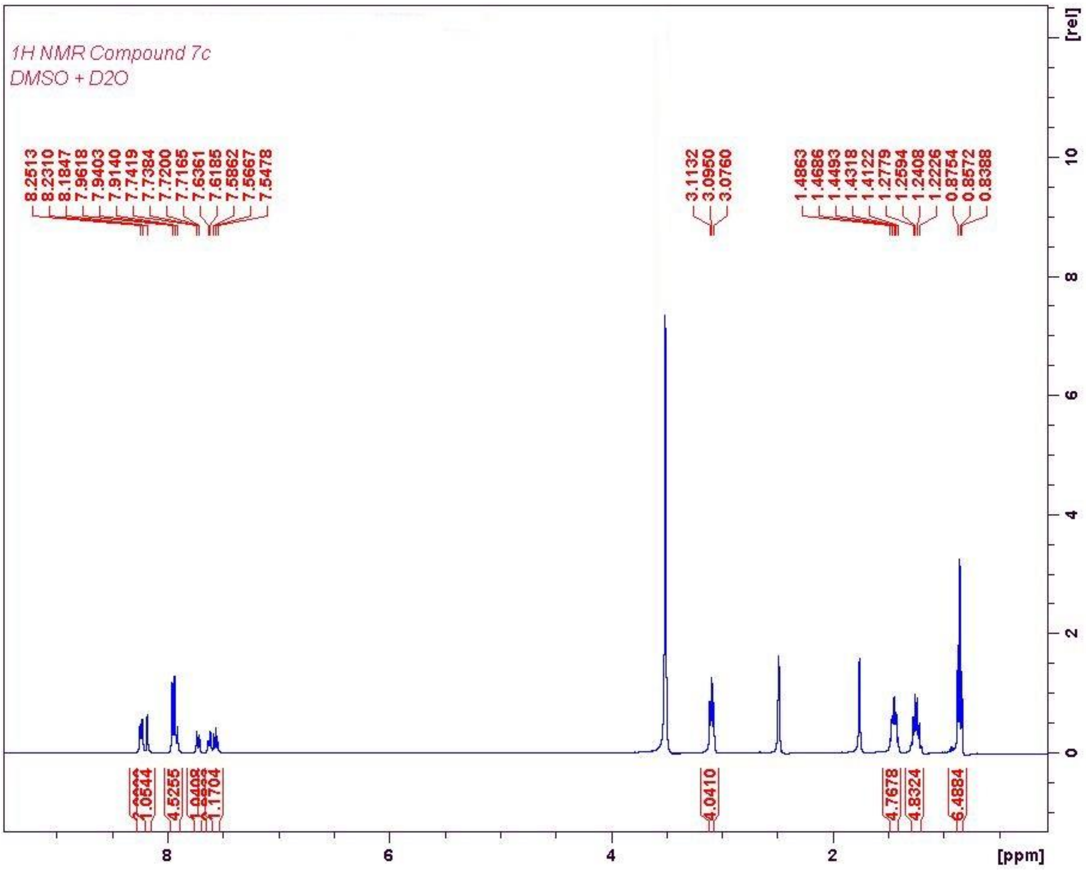

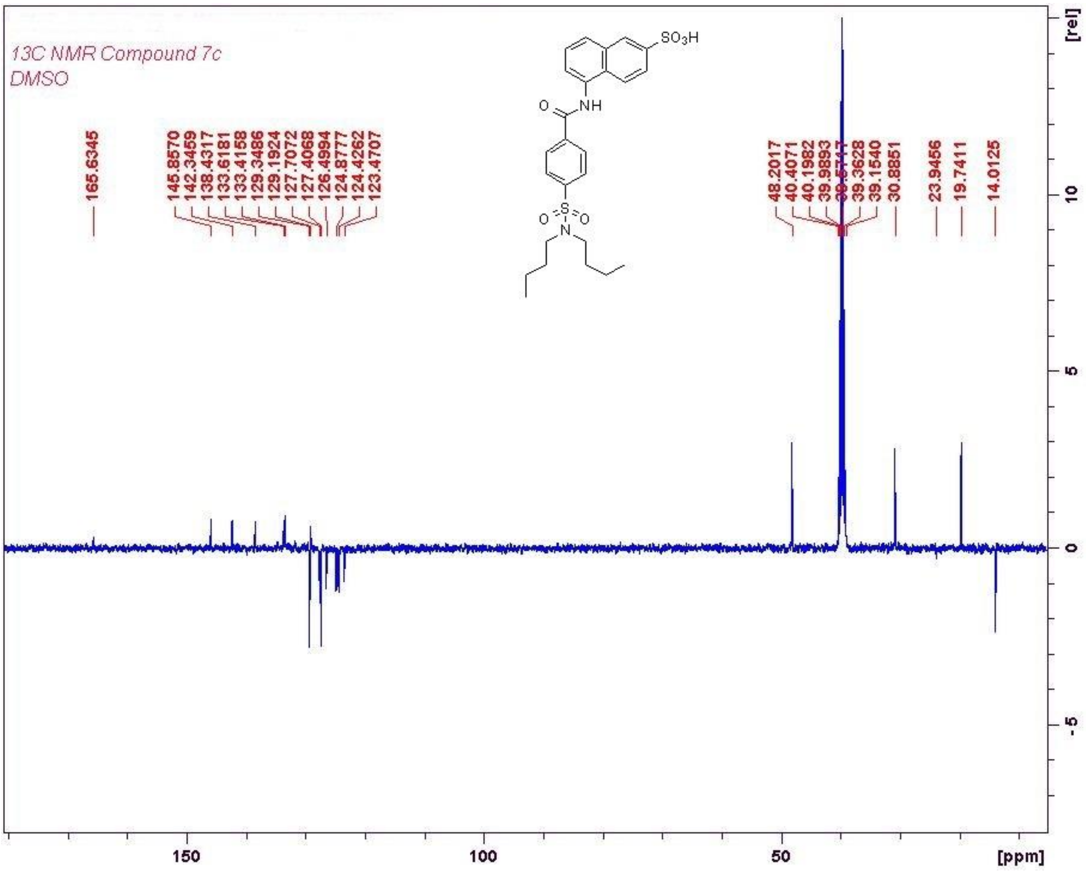
13C NMR Compound 7c
DMSO
SO3H
NH
O
O=S=O
N
165.6345
145.8570
142.3459
138.4317
133.6181
133.4158
129.3486
129.1924
127.7072
127.4068
126.4994
124.8777
124.4262
123.4707
48.2017
40.4071
40.1982
39.9893
39.3628
39.1540
30.8851
23.9456
19.7411
14.0125
[rel]
10
5
0
-5
150
100
50
[ppm]

^{1}H-NMR and ^{13}C-NMR spectra of compound **7d**

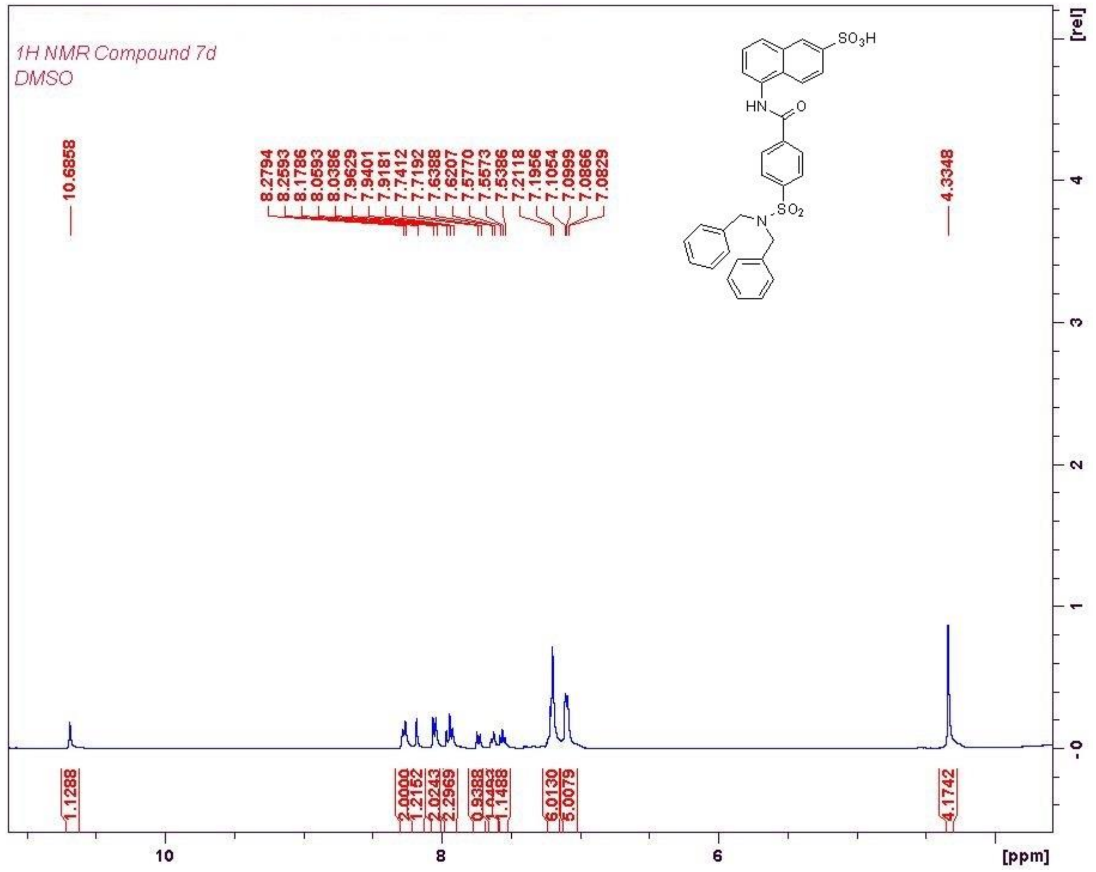

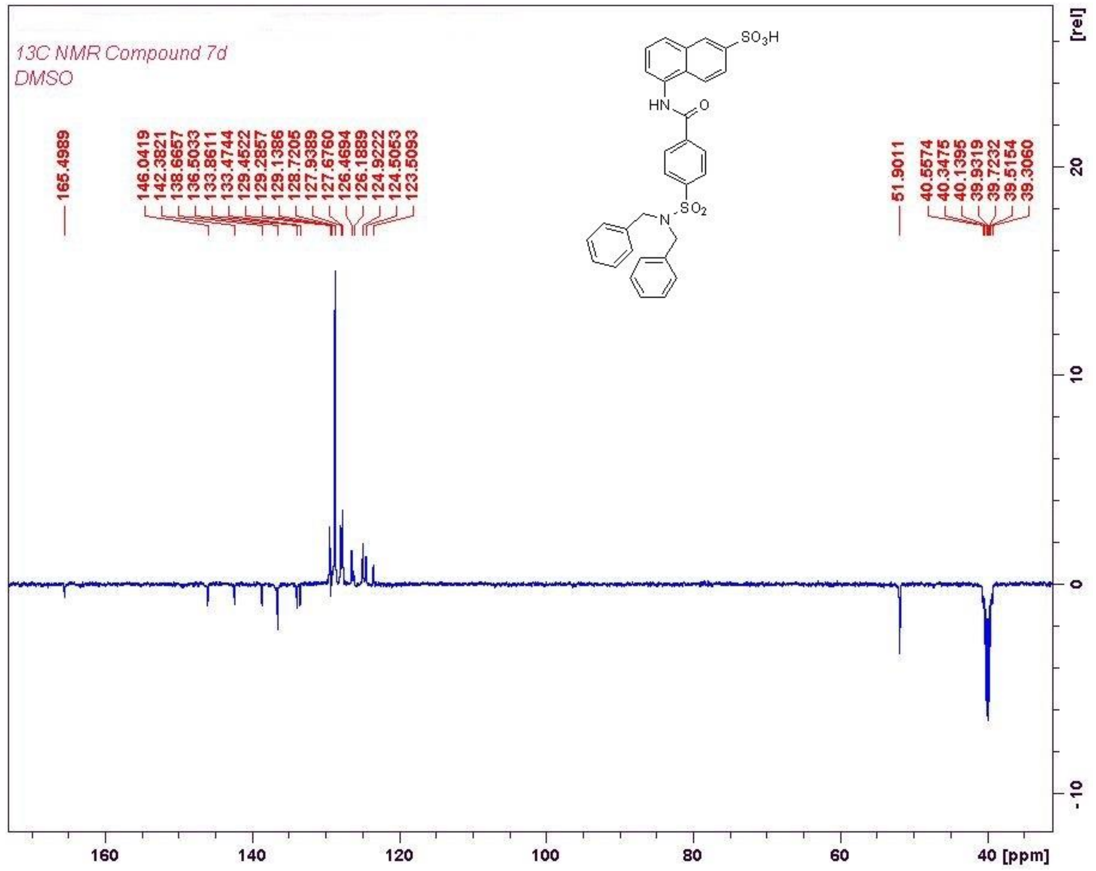

^{1}H-NMR and ^{13}C-NMR spectra of compound **7e**

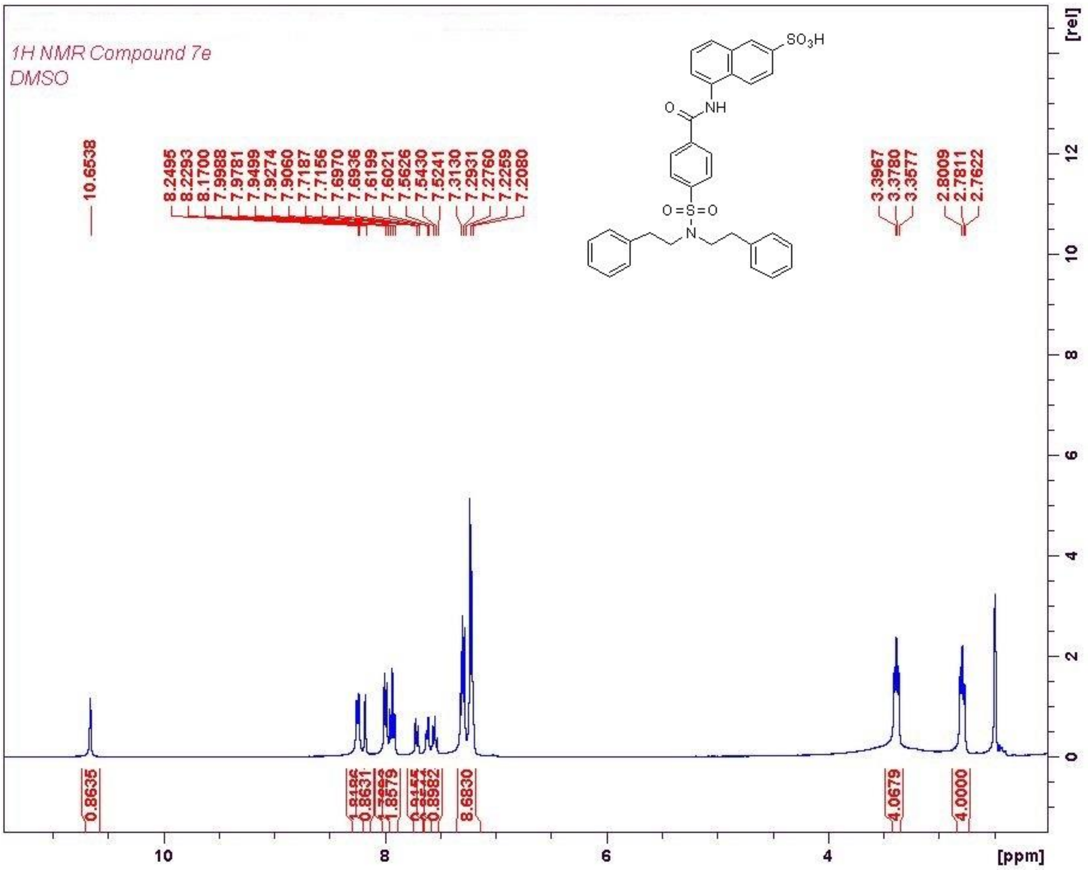

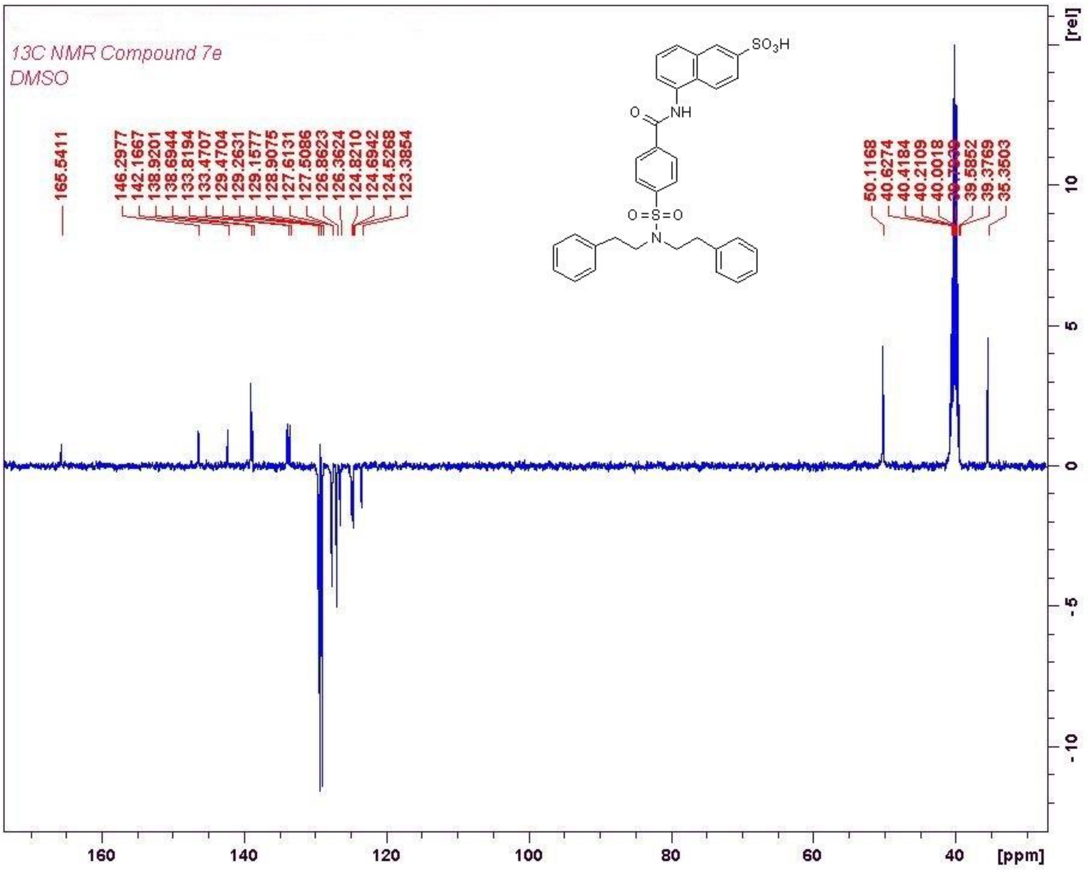

^{1}H-NMR and ^{13}C-NMR spectra of compound **7f**

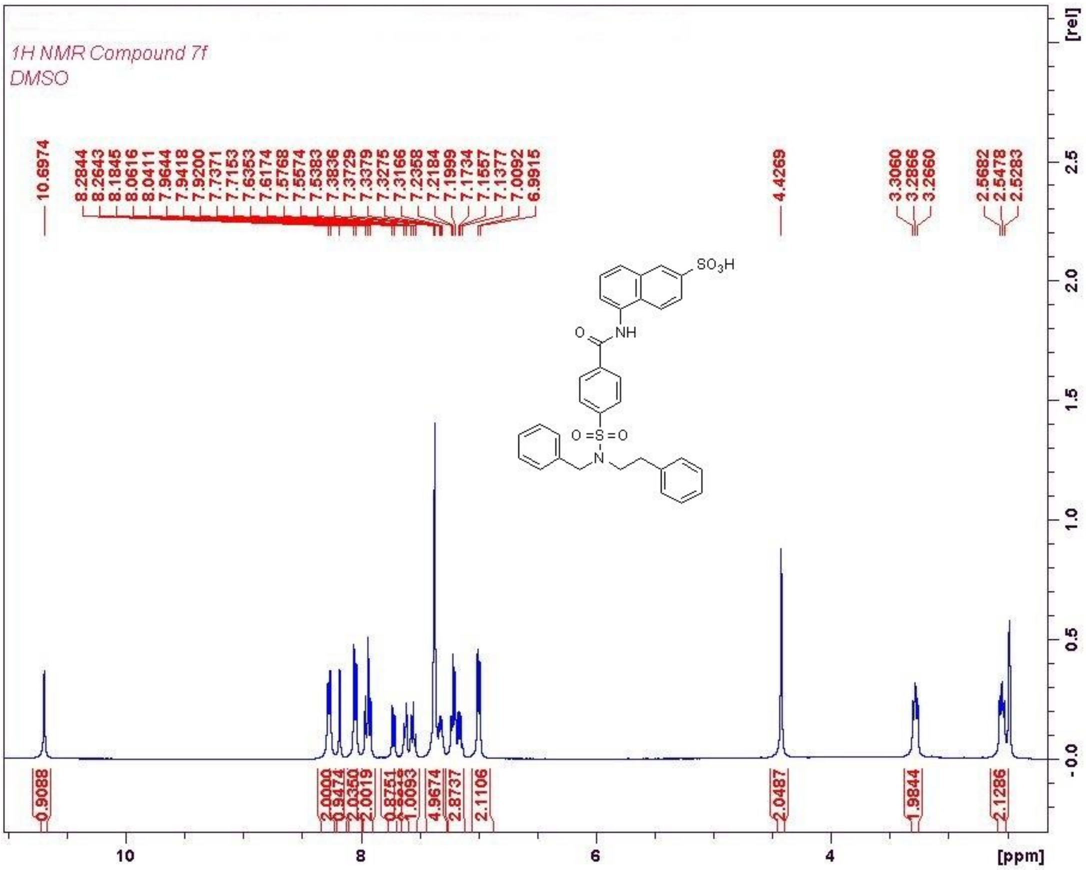

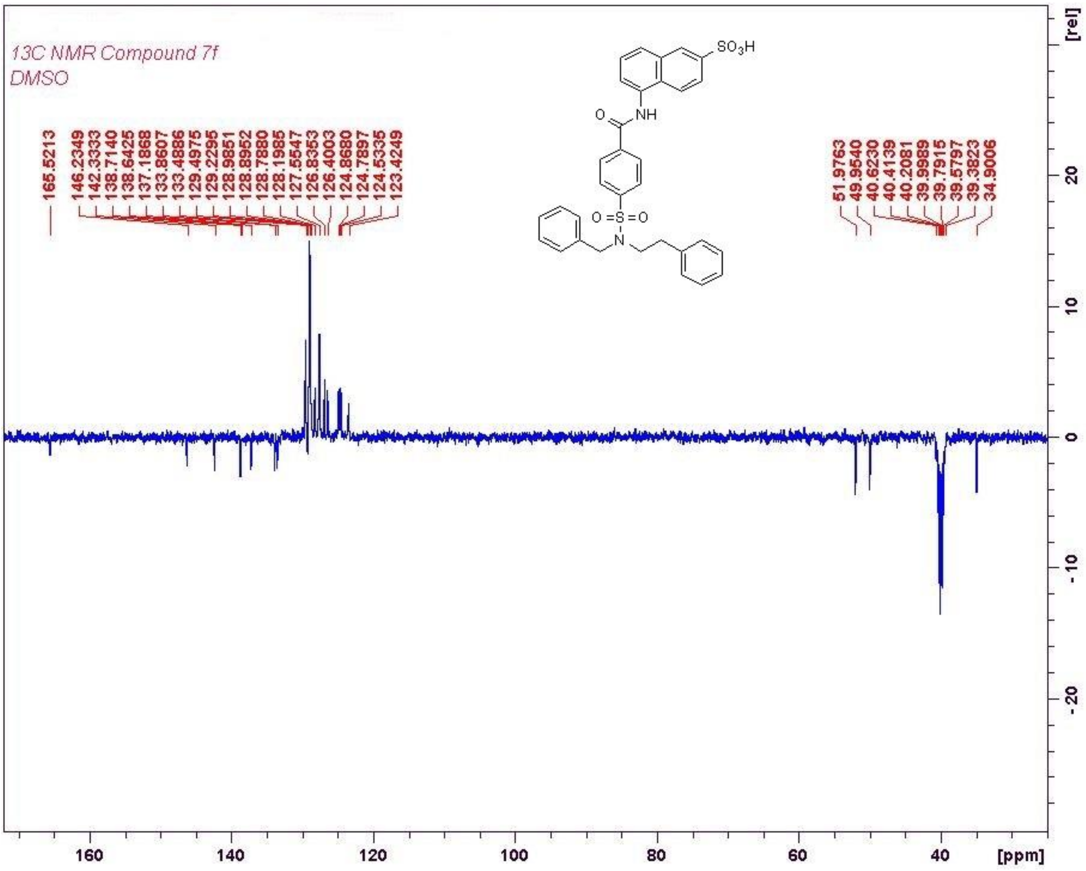

^{1}H-NMR and ^{13}C-NMR spectra of compound **7g**

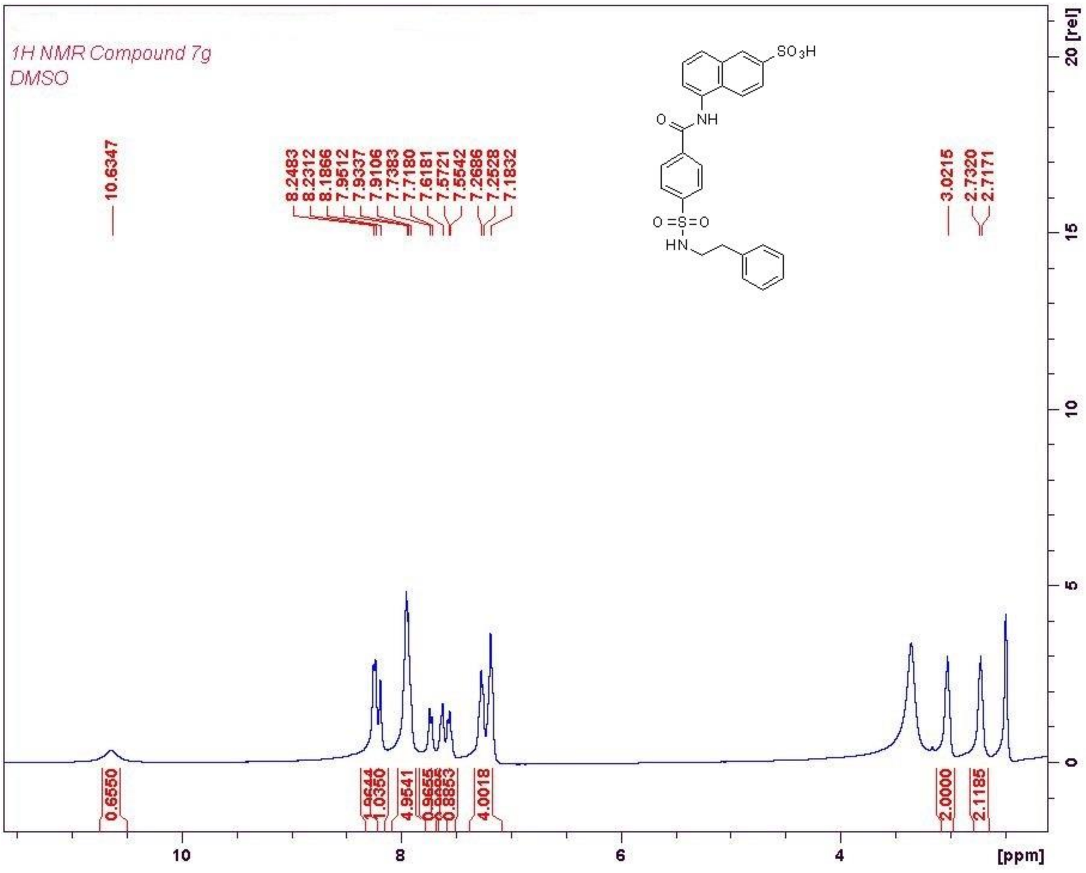

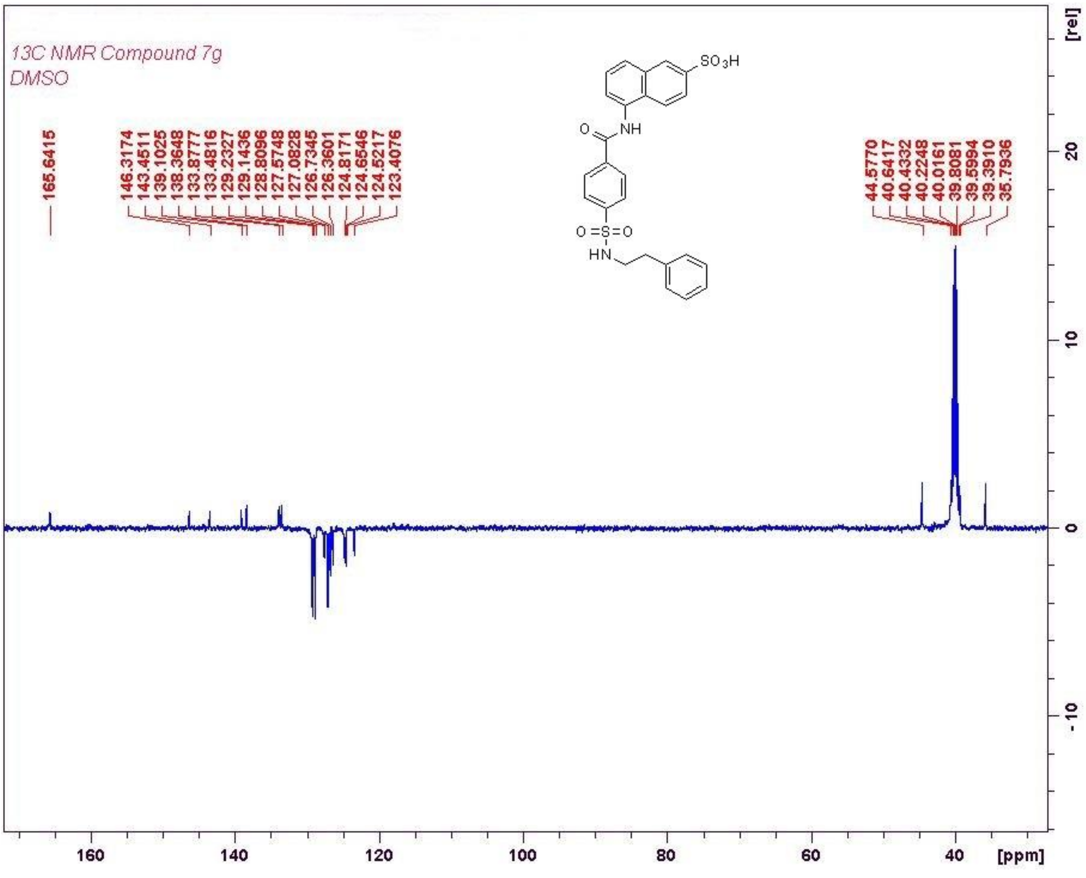

^{1}H-NMR and ^{13}C-NMR spectra of compound **13**

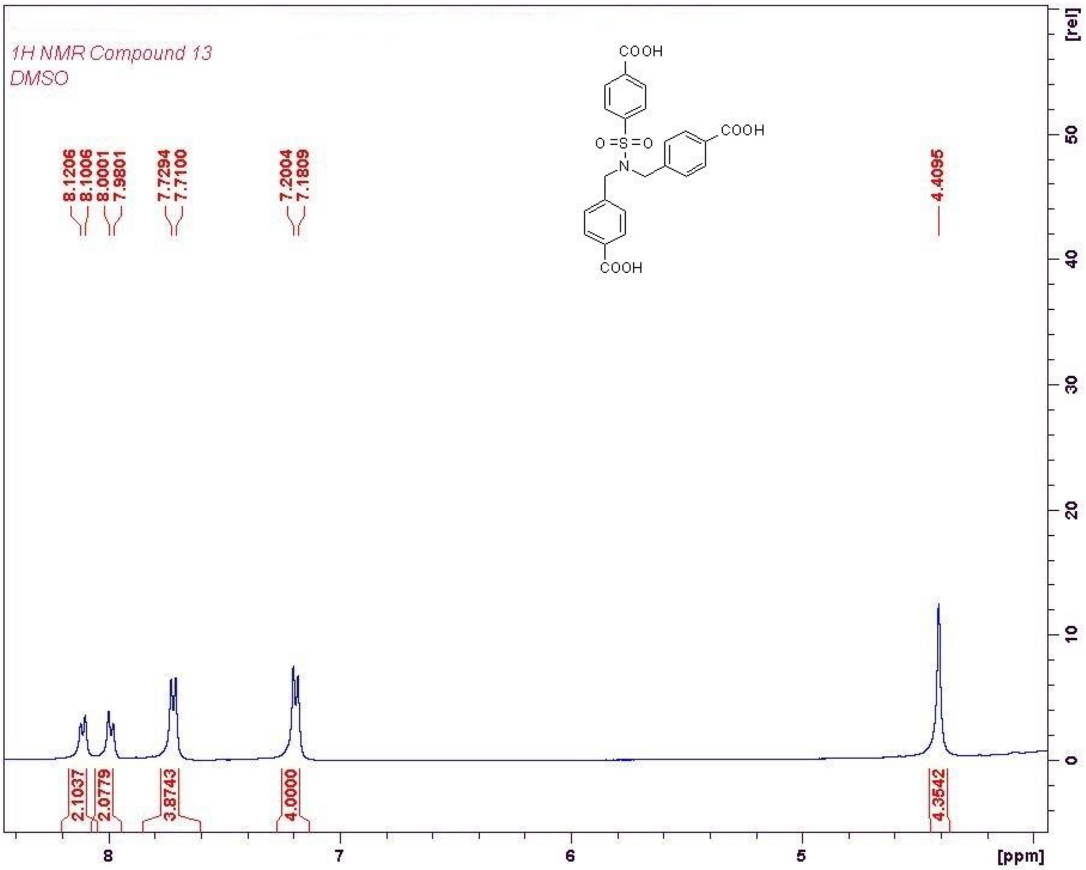

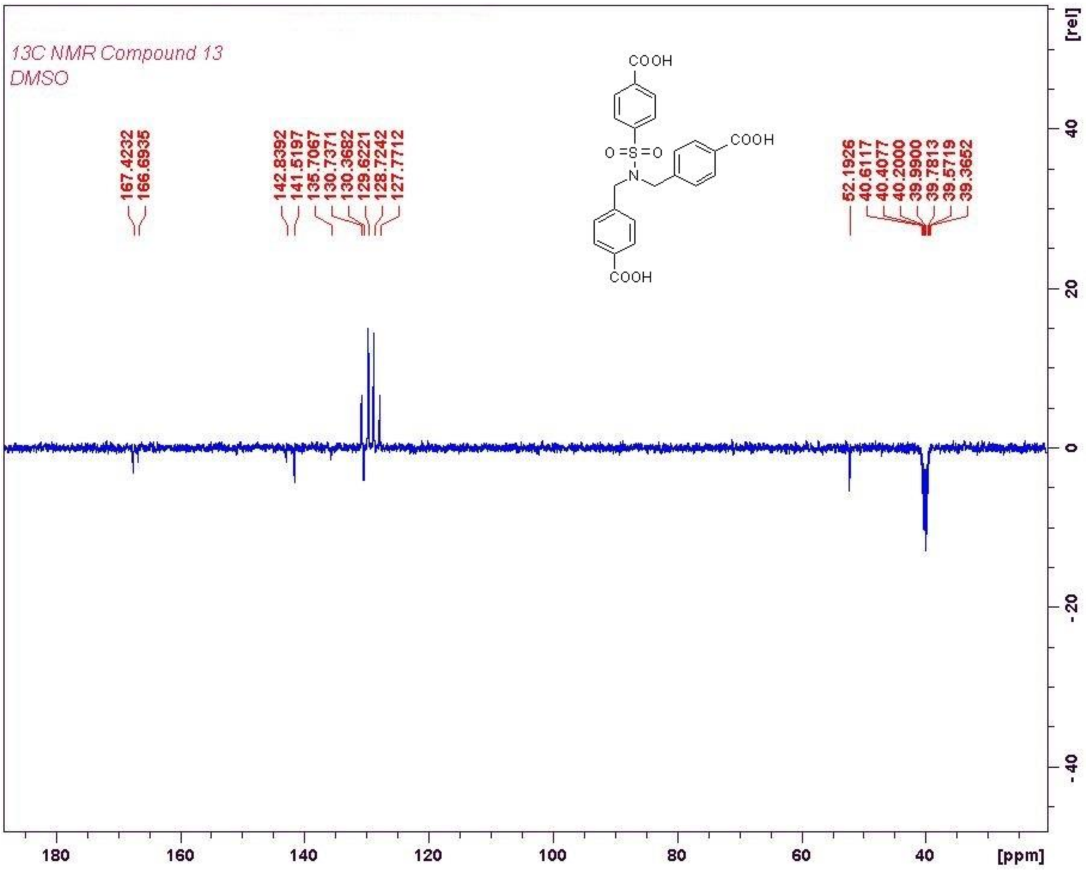

^{1}H-NMR spectrum of intermediate **15b**

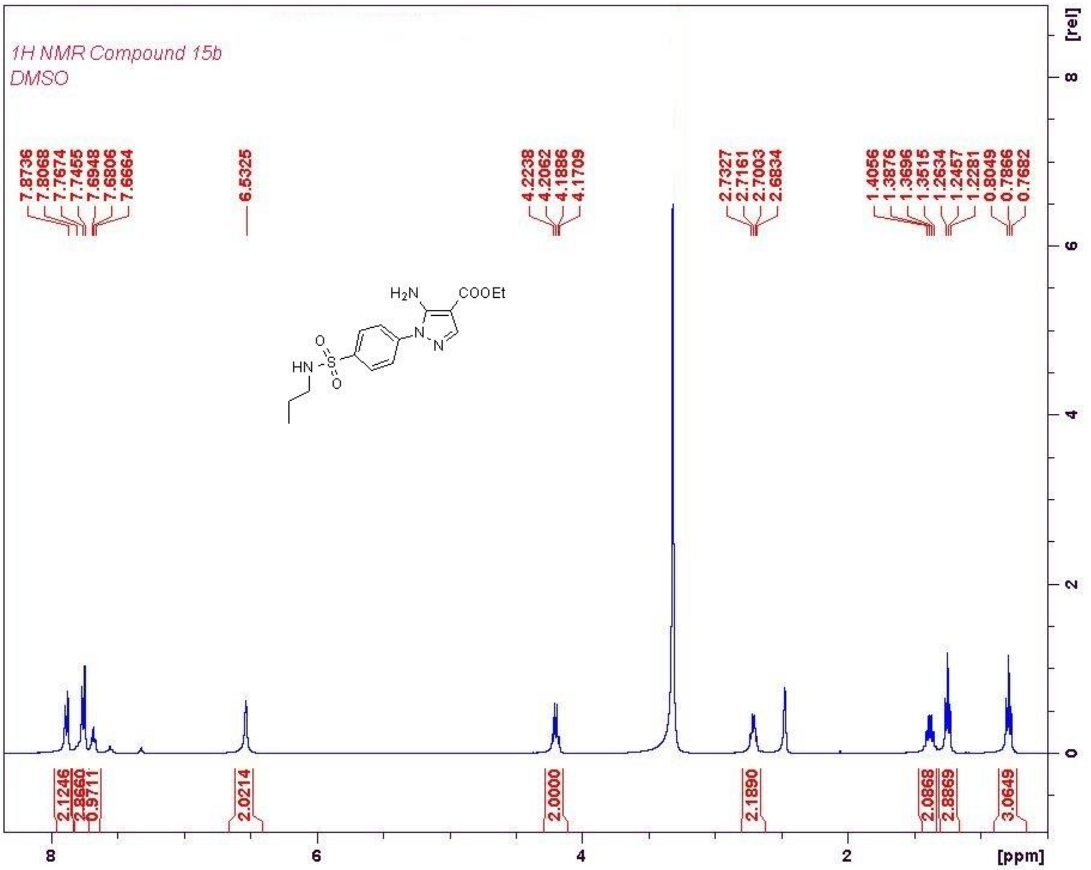

^{1}H-NMR spectrum of intermediate **15c**

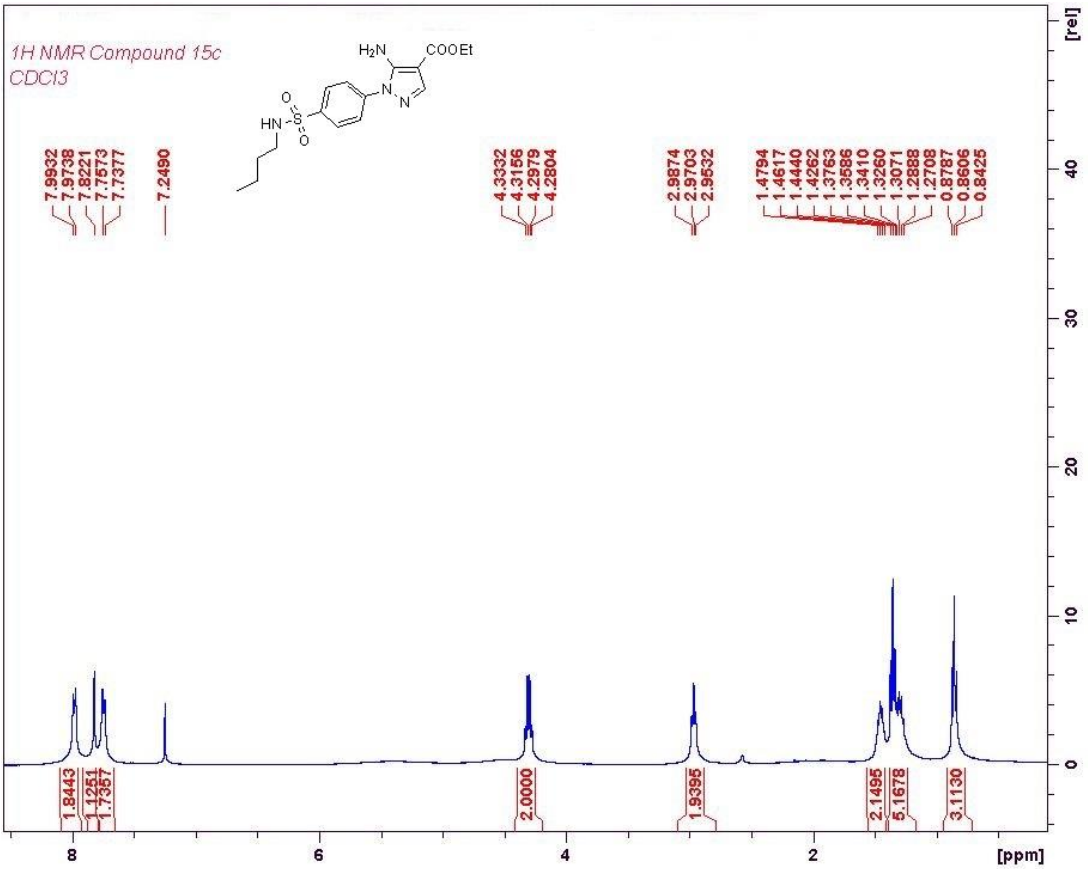

^{1}H-NMR spectrum of intermediate **16b**

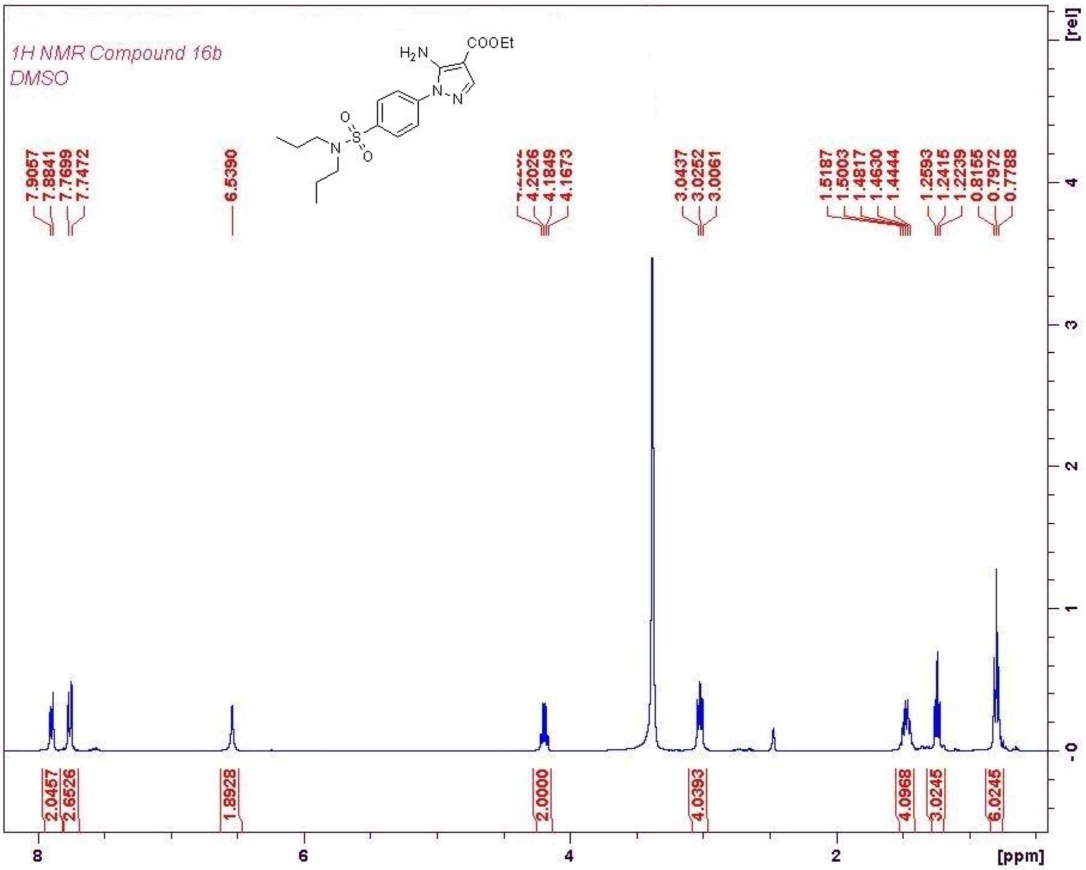

^{1}H-NMR spectrum of intermediate **16c**

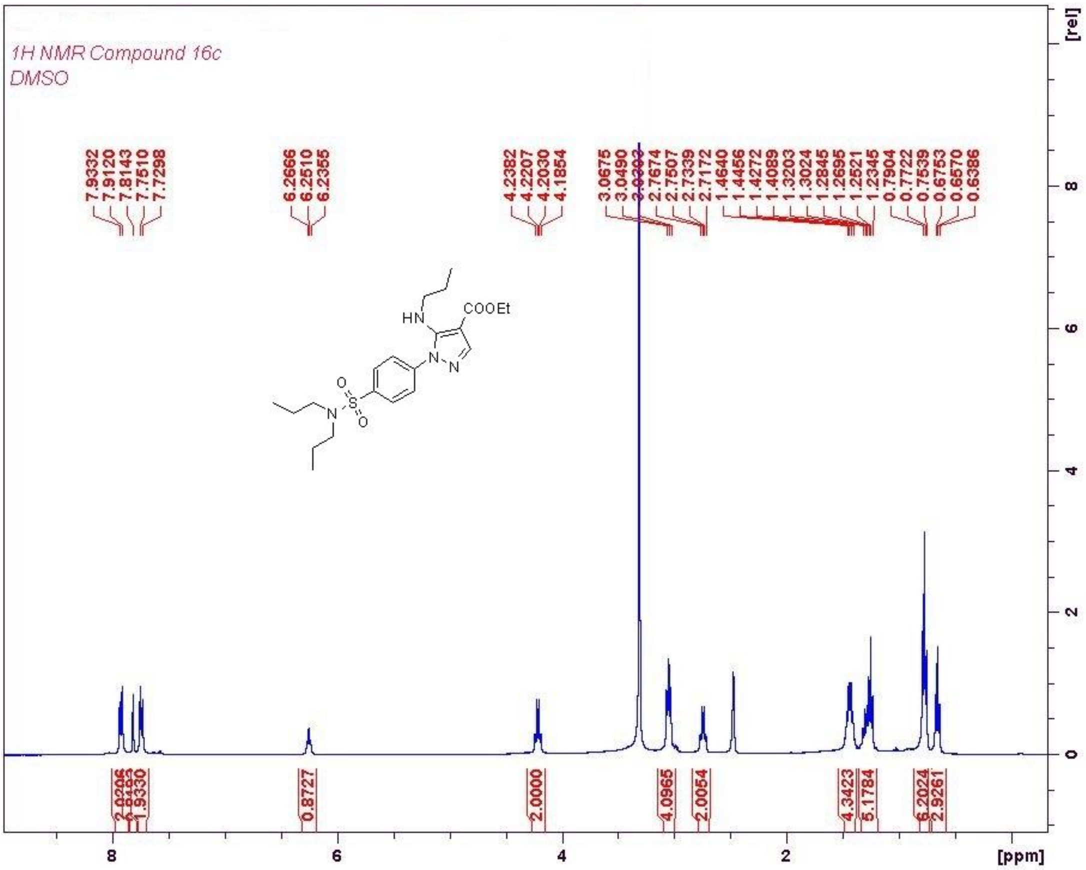

^{1}H-NMR and ^{13}C-NMR spectra of compound **17**

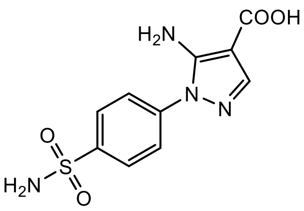

1H NMR Compound 17
DMSO

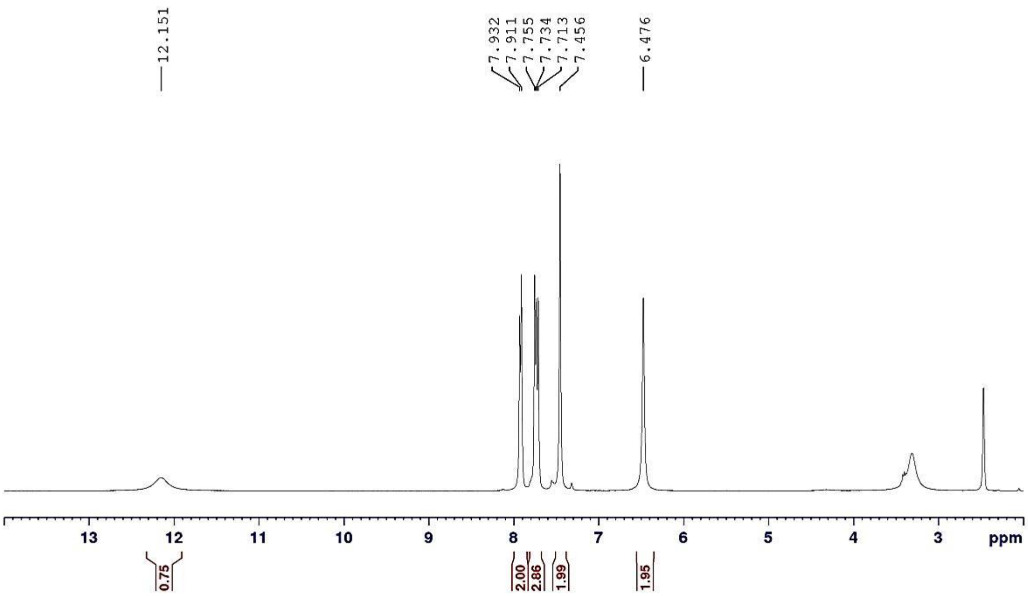

13C NMR Compound 17
DMSO

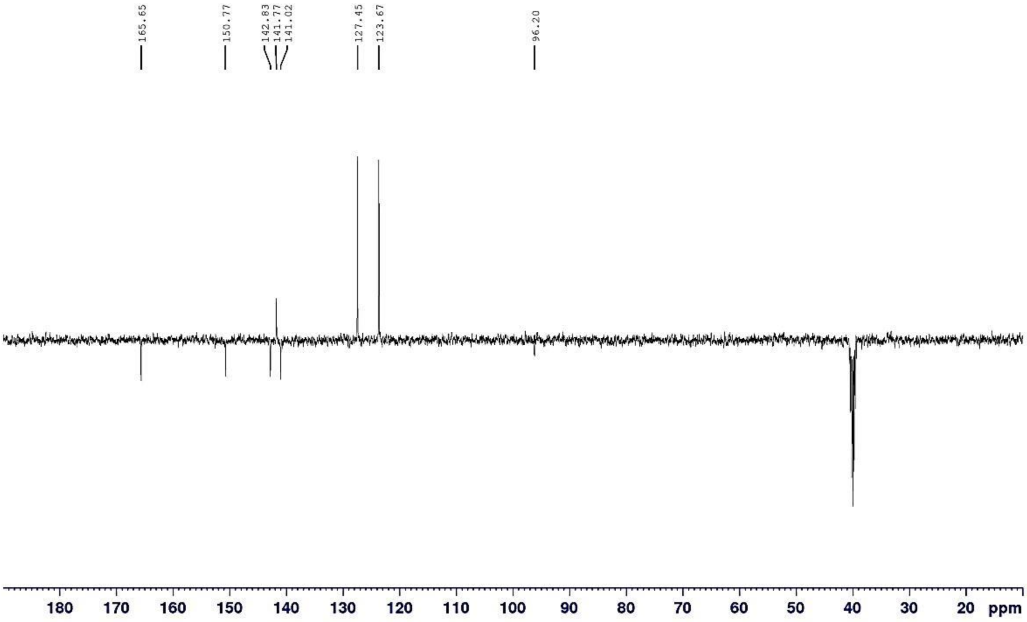

^{1}H-NMR and ^{13}C-NMR spectra of compound **18c**

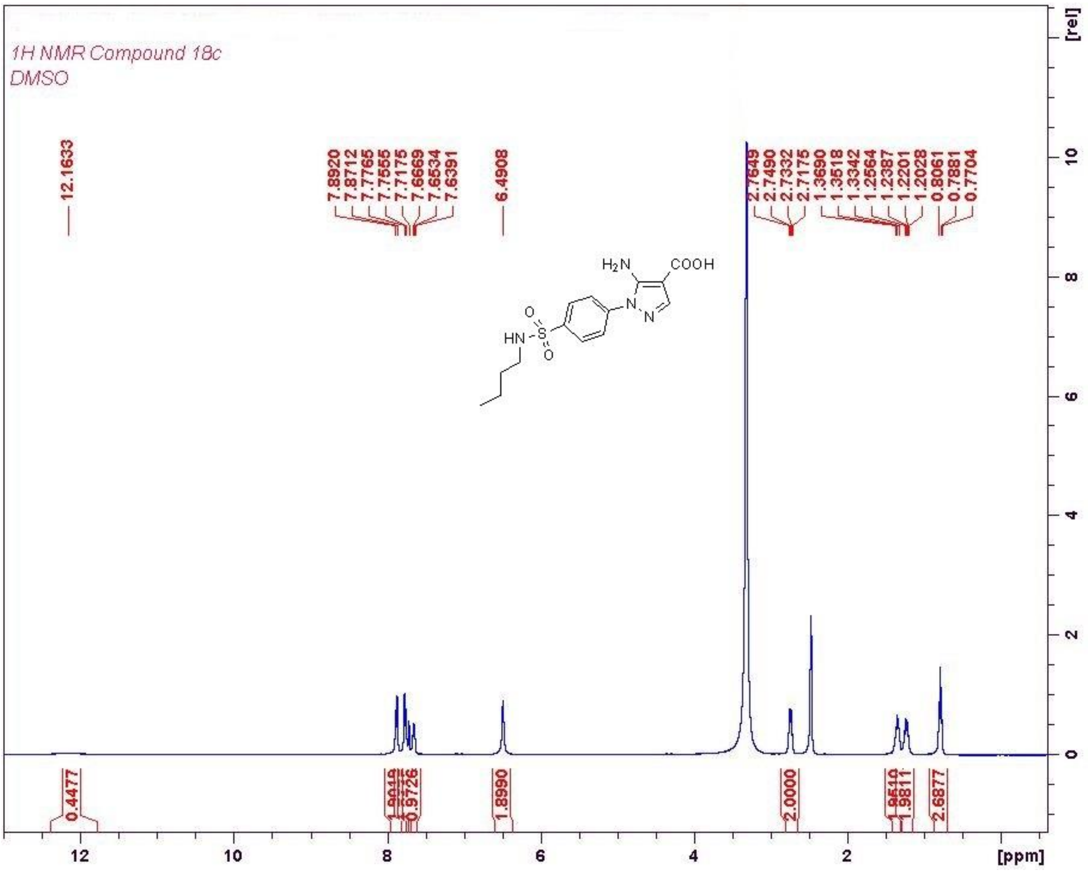

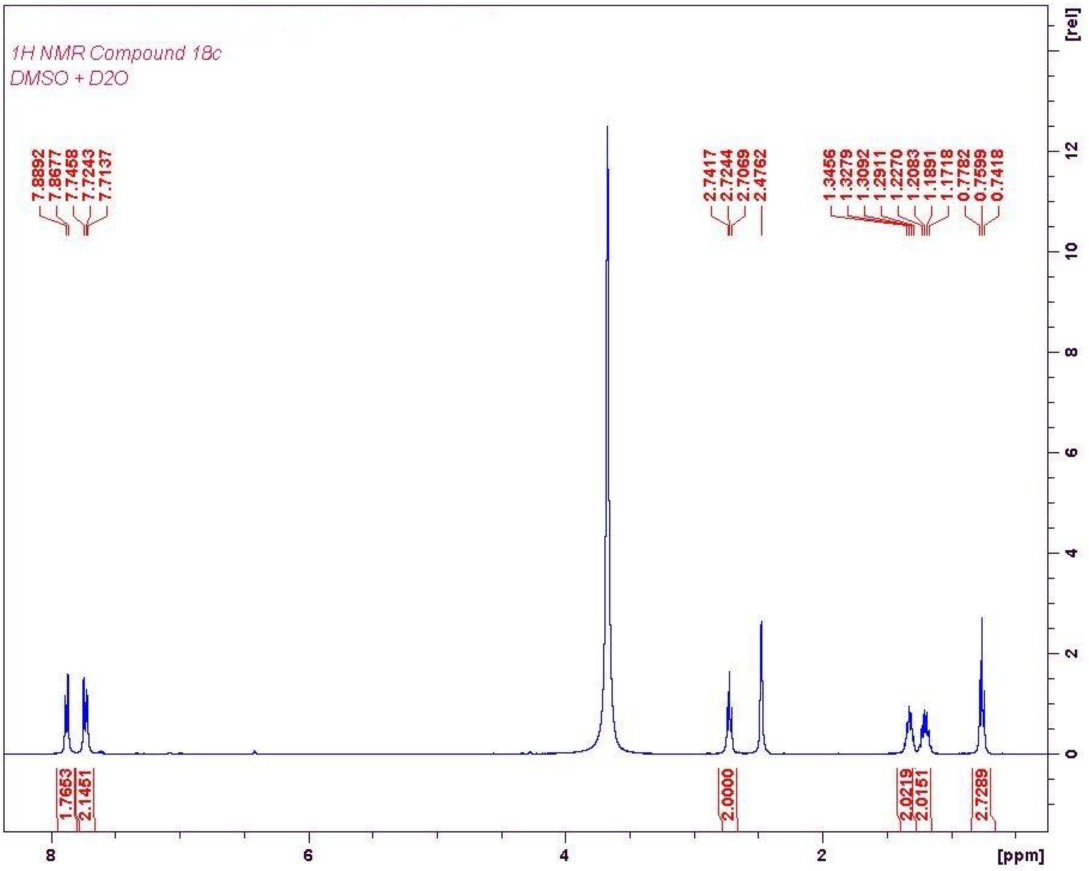

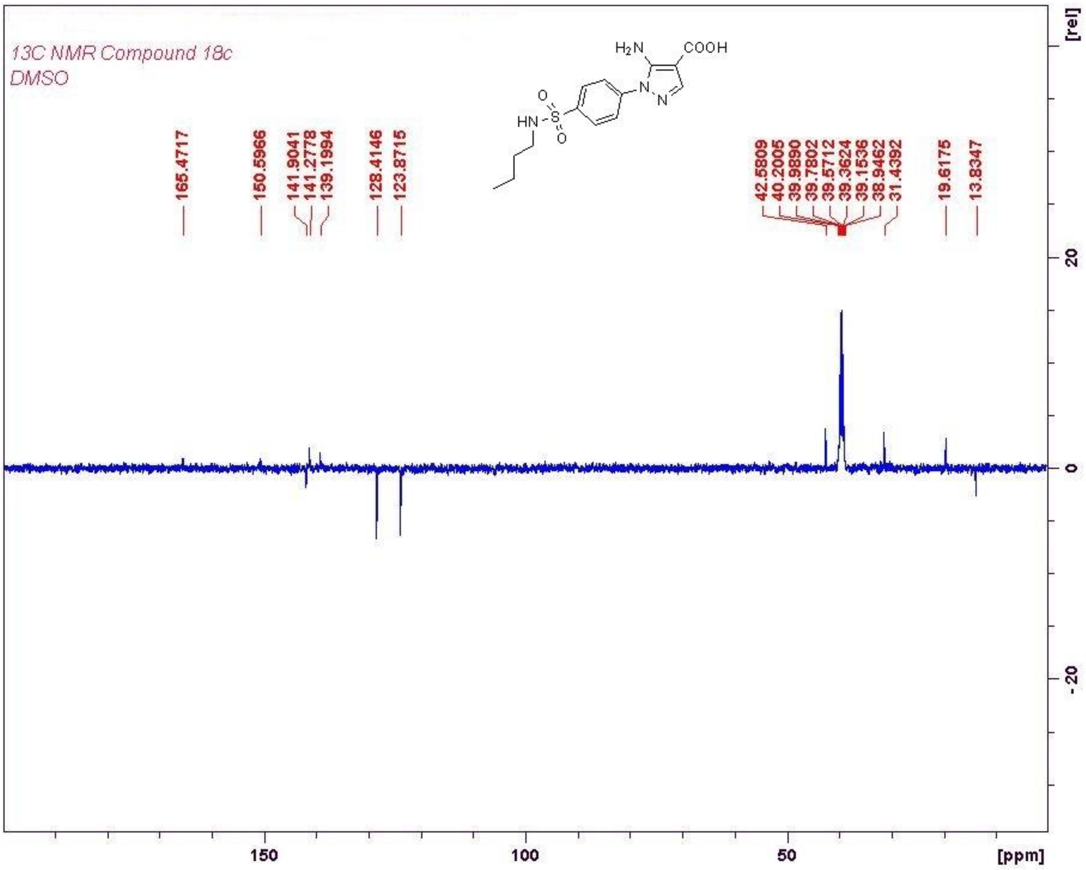
13C NMR Compound 18c
DMSO
165.4717
150.5966
141.9041
141.2778
139.1994
128.4146
123.8715
42.5809
40.2005
39.9890
39.7802
39.5712
39.3624
39.1536
38.9462
31.4392
19.6175
13.8347
[rel]
20
0
-20
150
100
50
[ppm]

^{1}H-NMR and ^{13}C-NMR spectra of compound **19b**

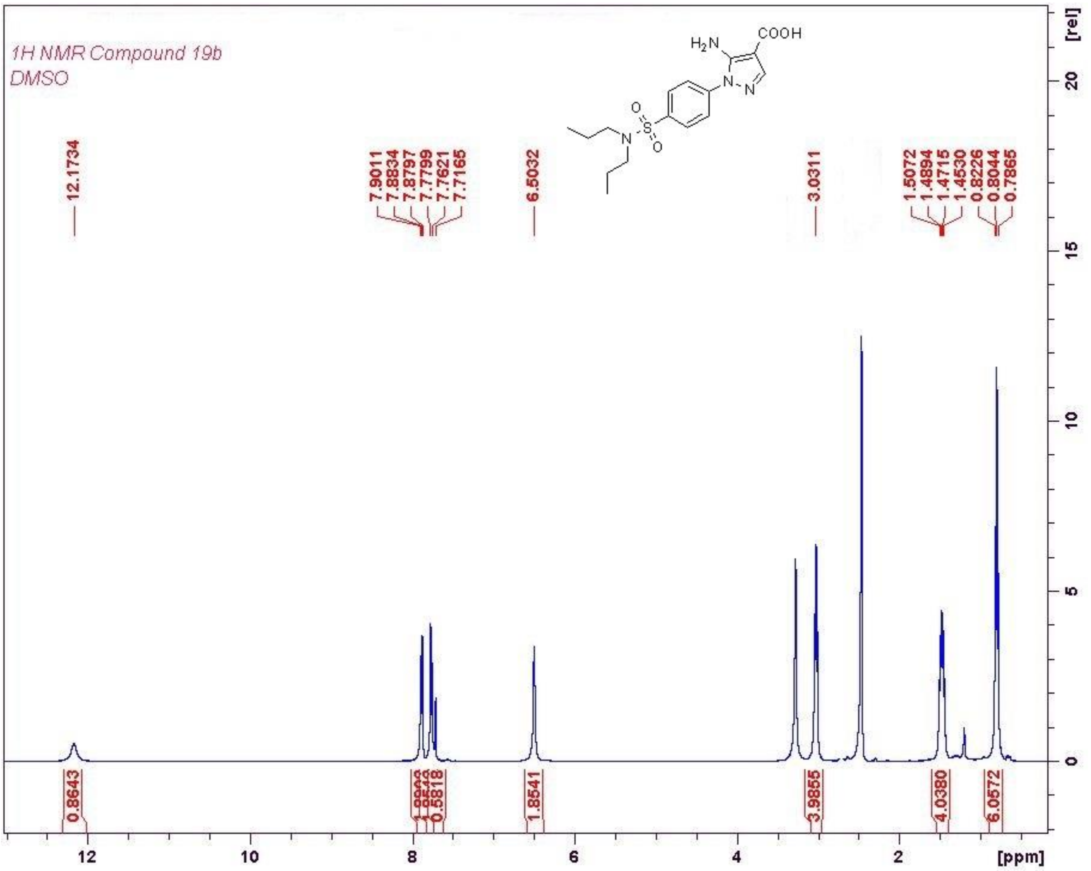

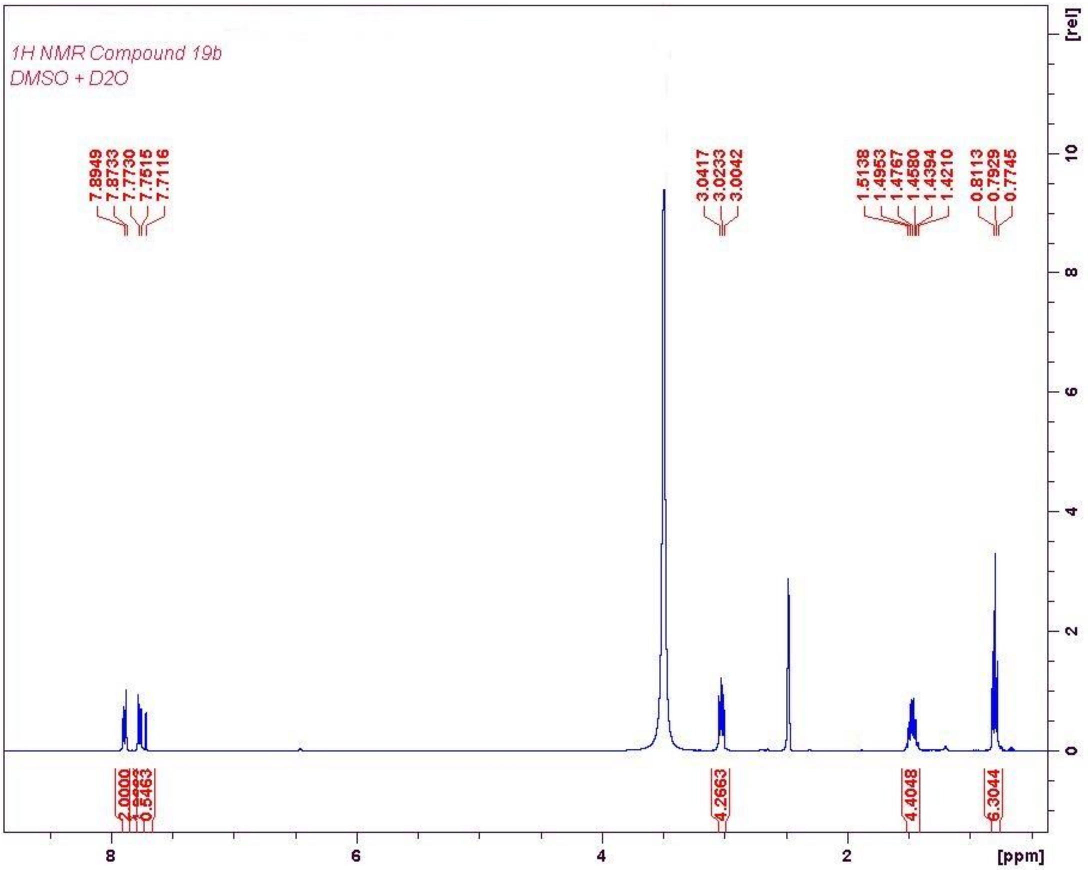

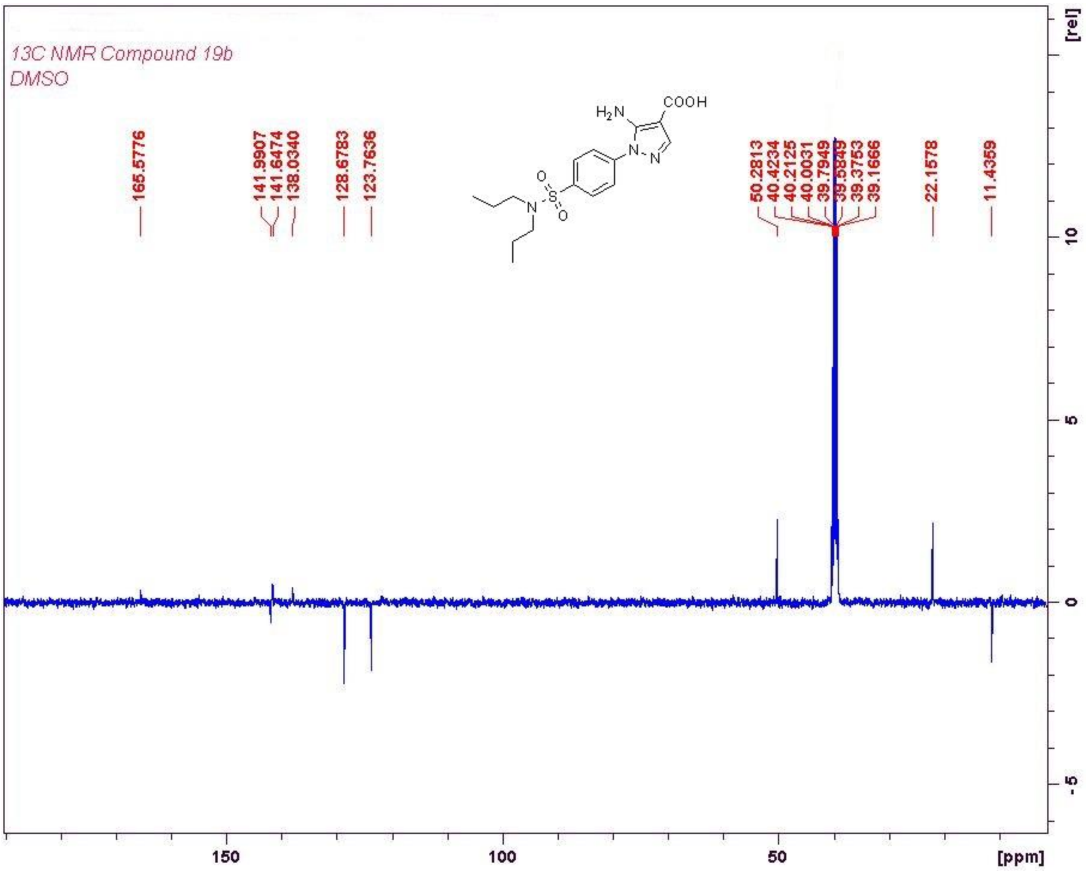

13C NMR Compound 19b
DMSO
165.5776
141.9907
141.6474
138.0340
128.6783
123.7636
50.2813
40.4234
40.2125
40.0031
39.7949
39.5849
39.3753
39.1666
22.1578
11.4359
[rel]
[ppm]

^{1}H-NMR and ^{13}C-NMR spectra of compound **19c**

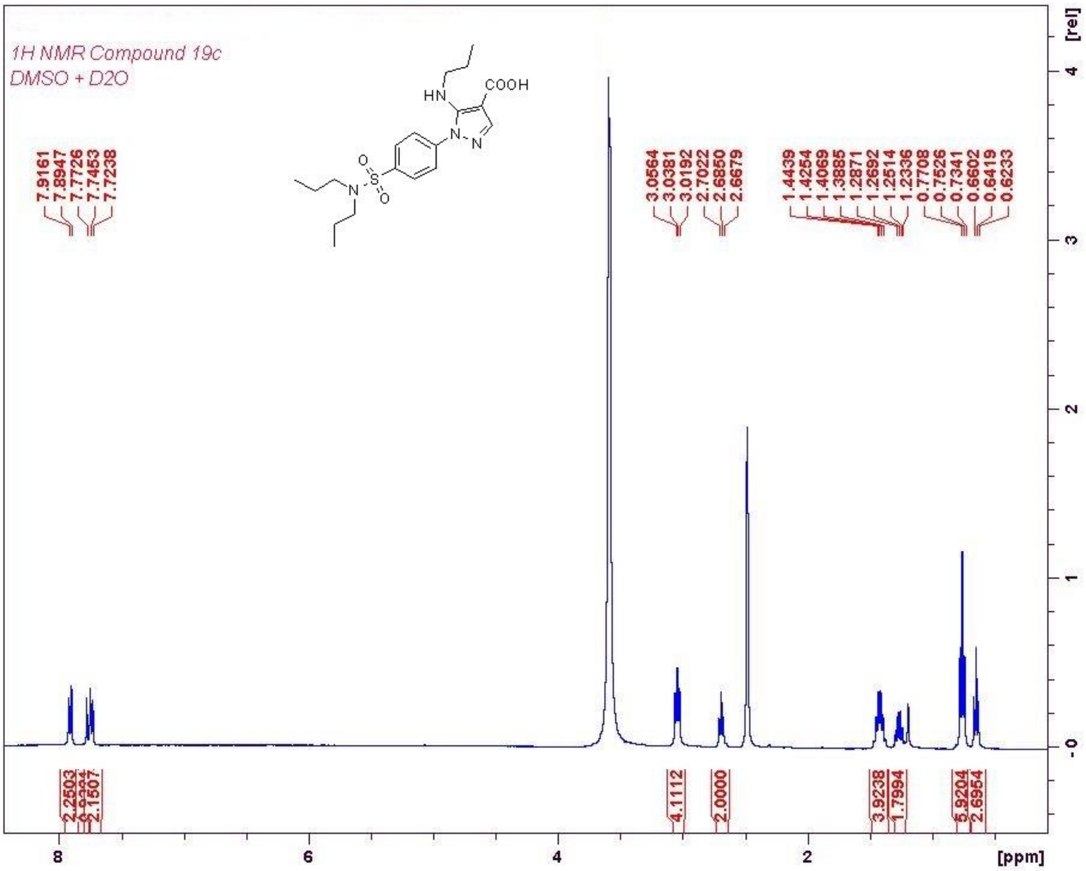

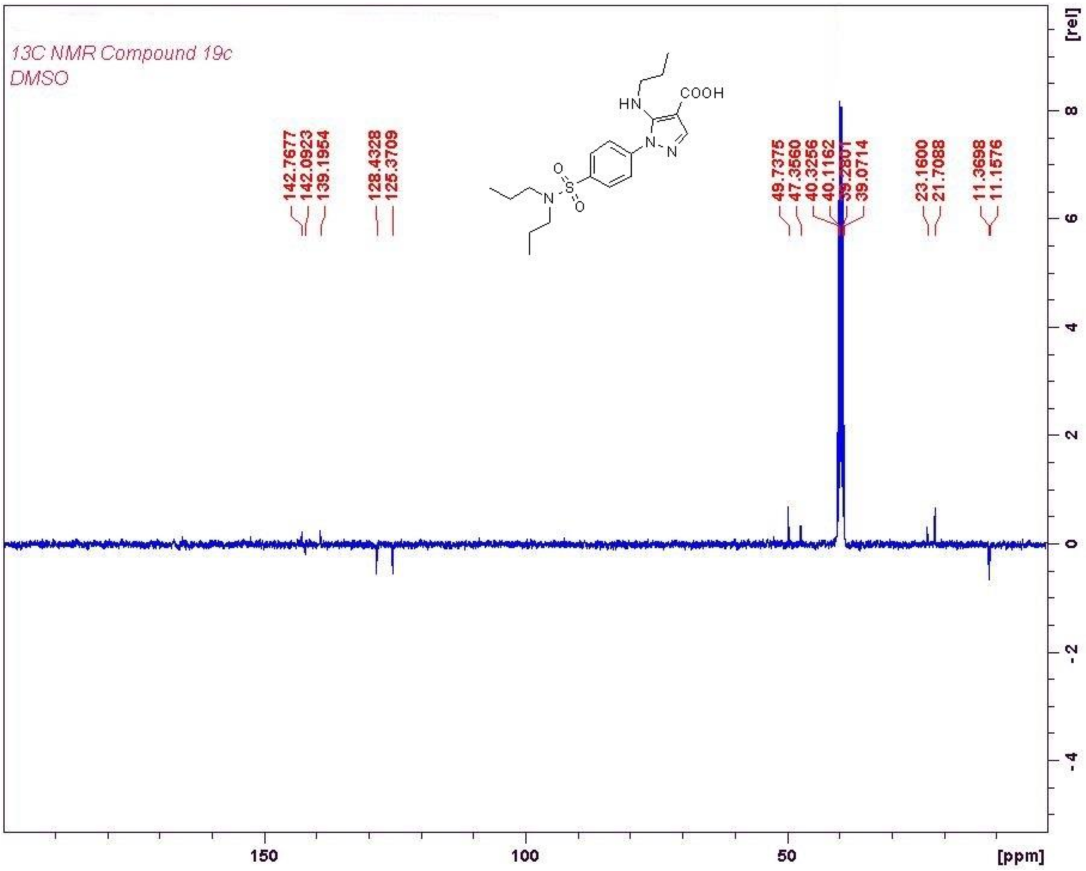

^{1}H-NMR and ^{13}C-NMR spectra of compound **24**

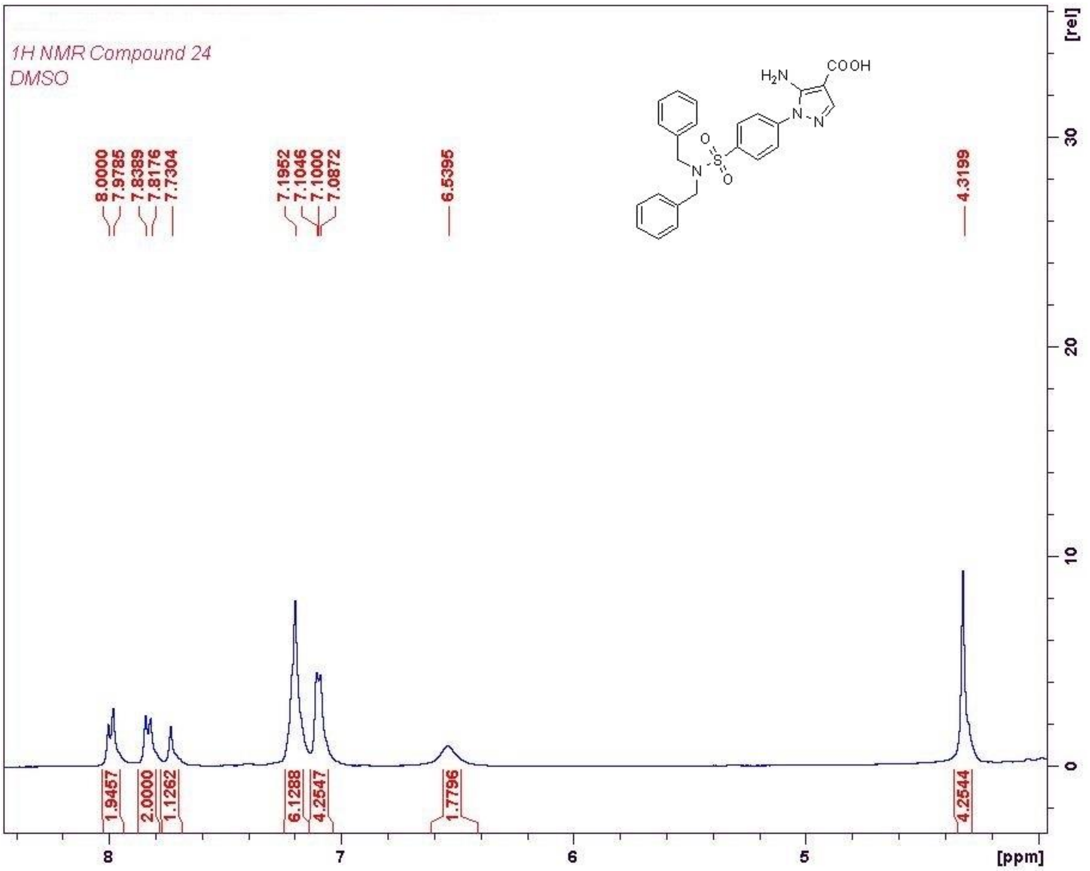

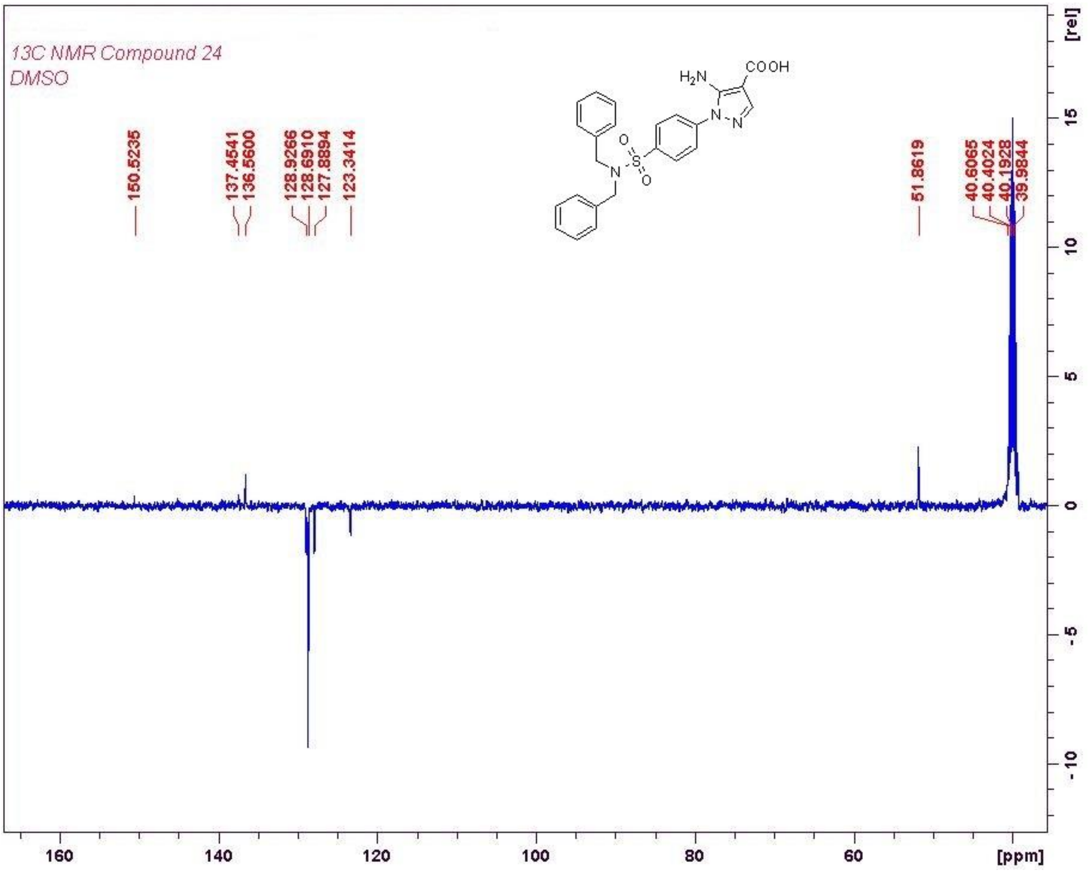

^{1}H-NMR spectrum of intermediate **25**

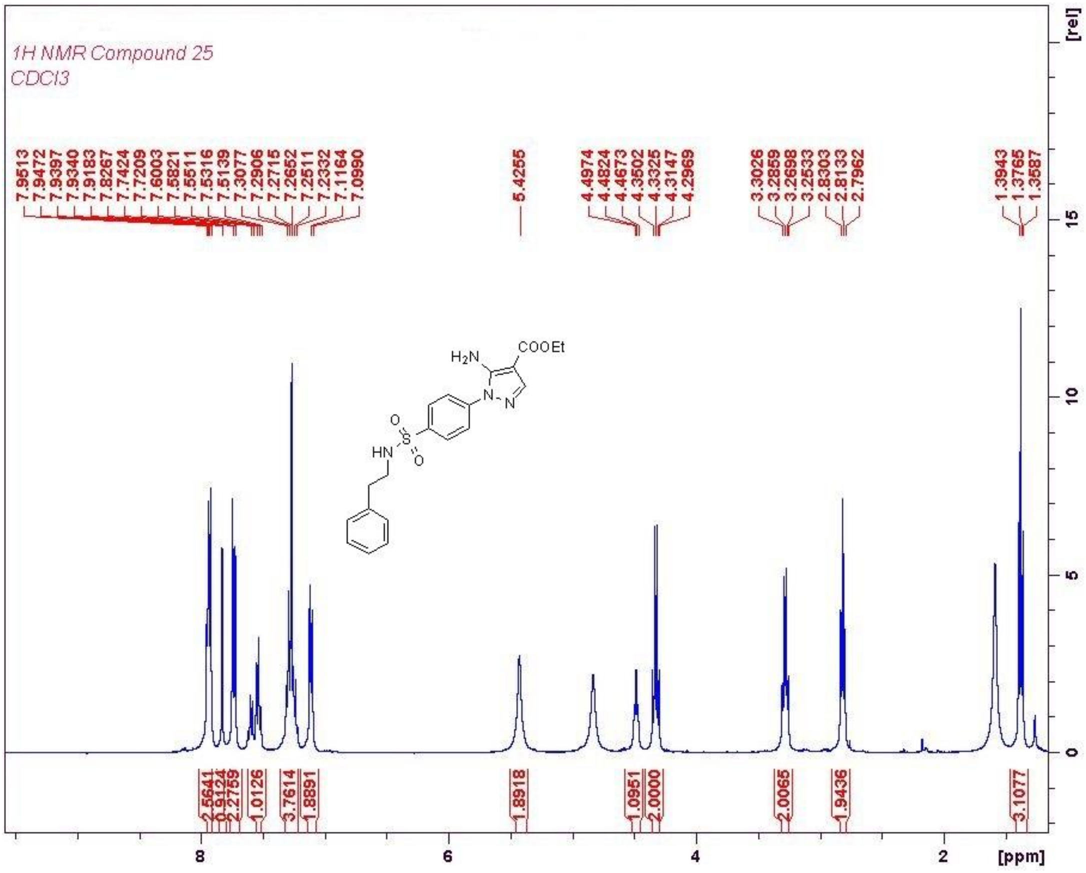

^{1}H-NMR and ^{13}C-NMR spectra of compound **28**

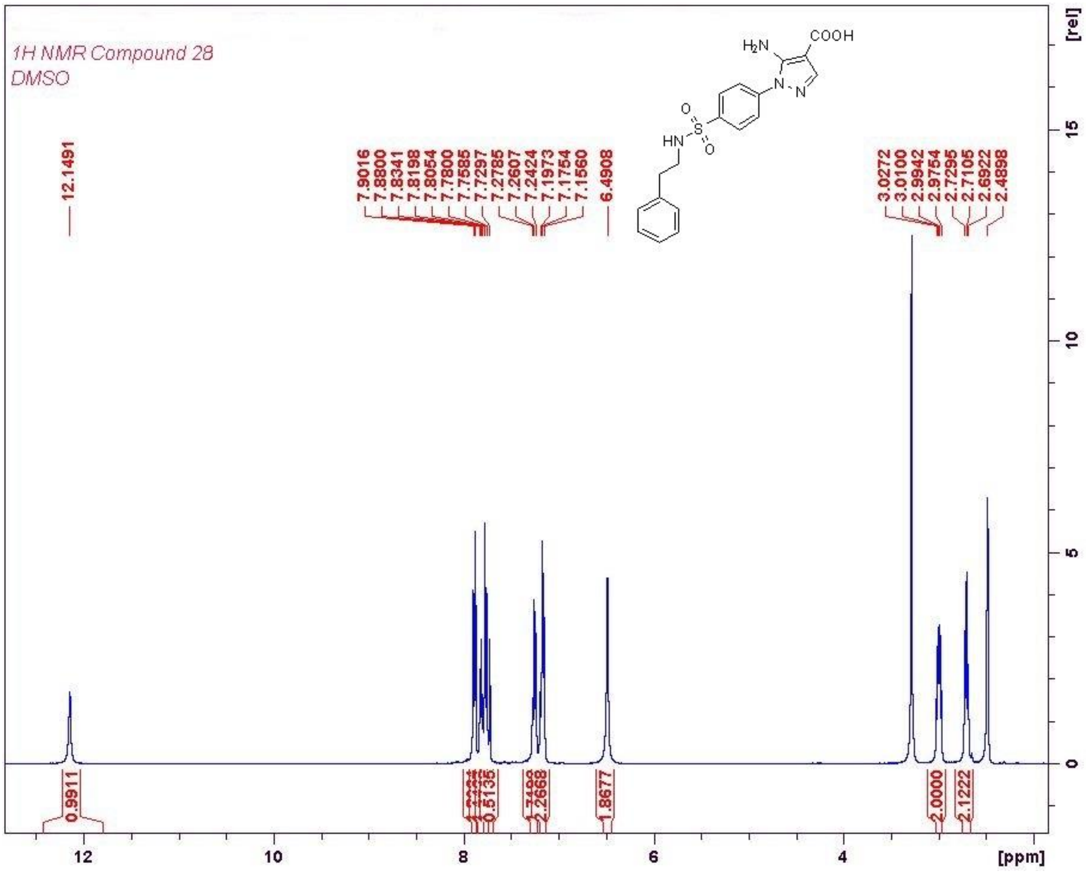

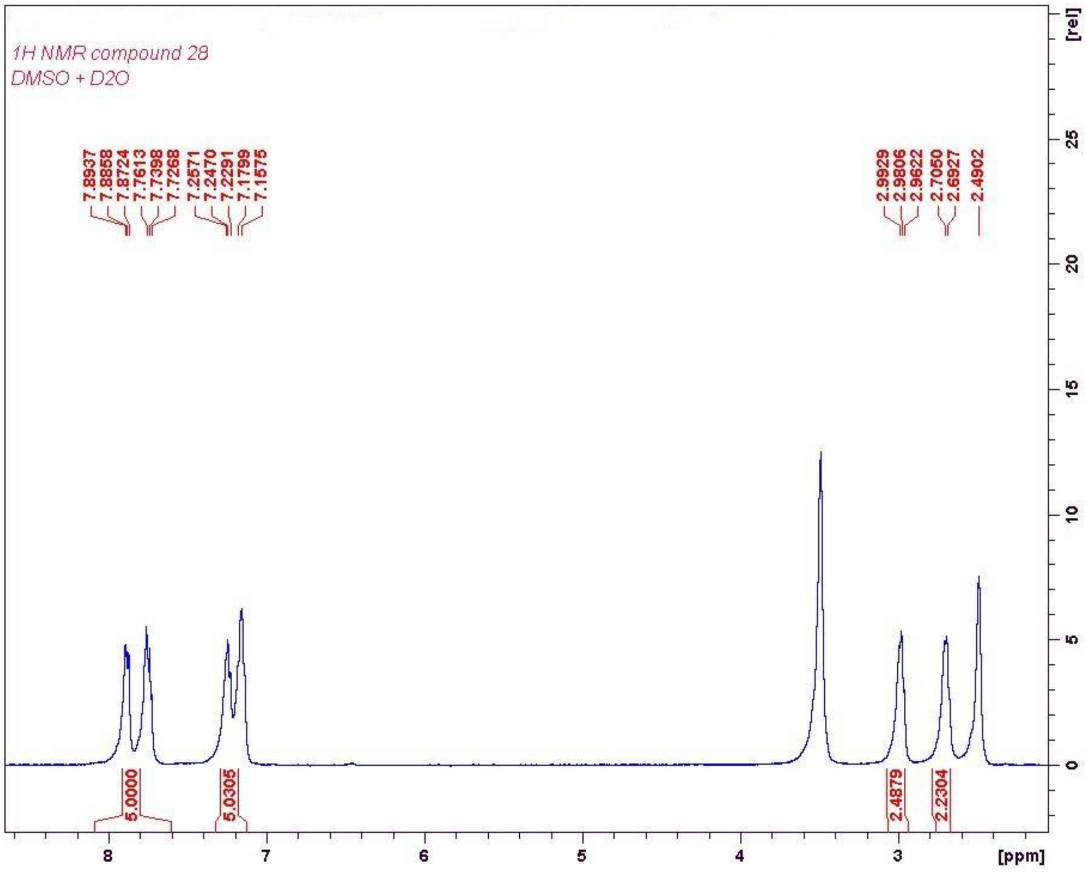

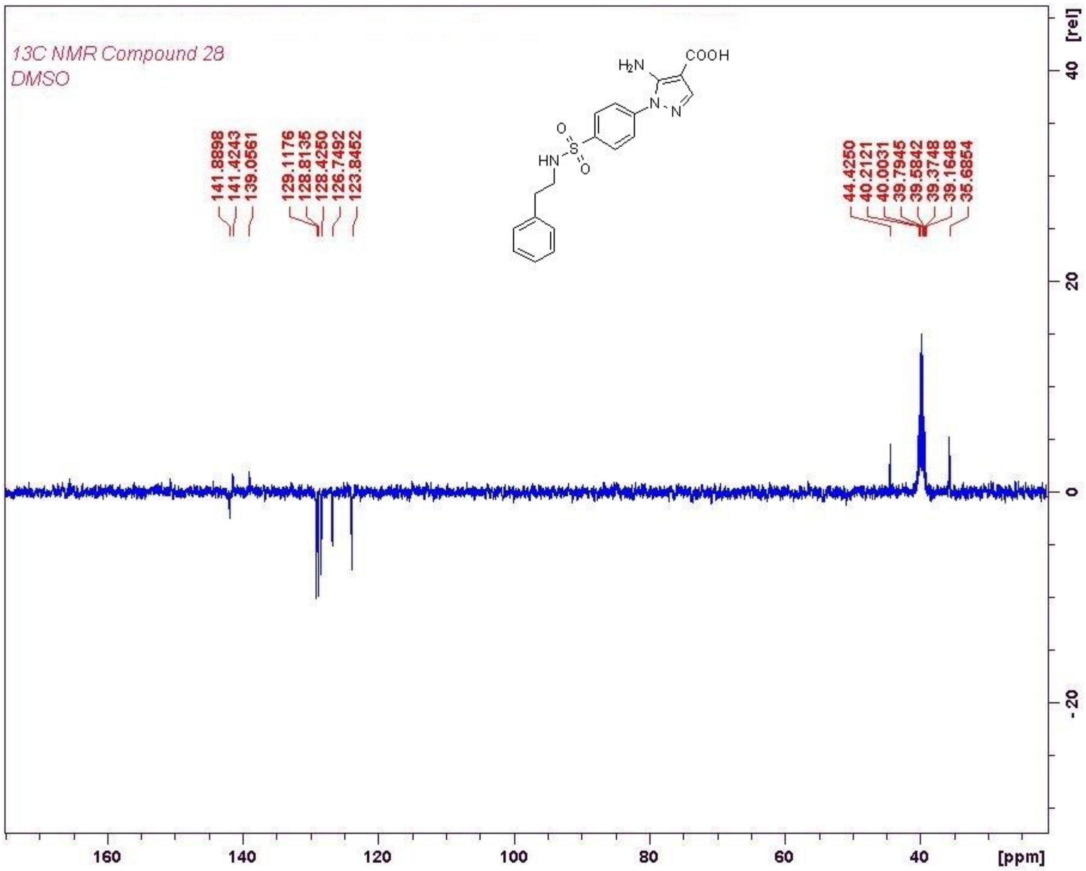

13C NMR Compound 28
DMSO
141.8898
141.4243
139.0561
129.1176
128.8135
128.4250
126.7492
123.8452
44.4250
40.2121
40.0031
39.7945
39.5842
39.3748
39.1648
35.6854
H2N
COOH
N
N
O
S
O
HN
[rel]
40
20
0
-20
160
140
120
100
80
60
40
[ppm]

^{1}H-NMR and ^{13}C-NMR spectra of compound **30**

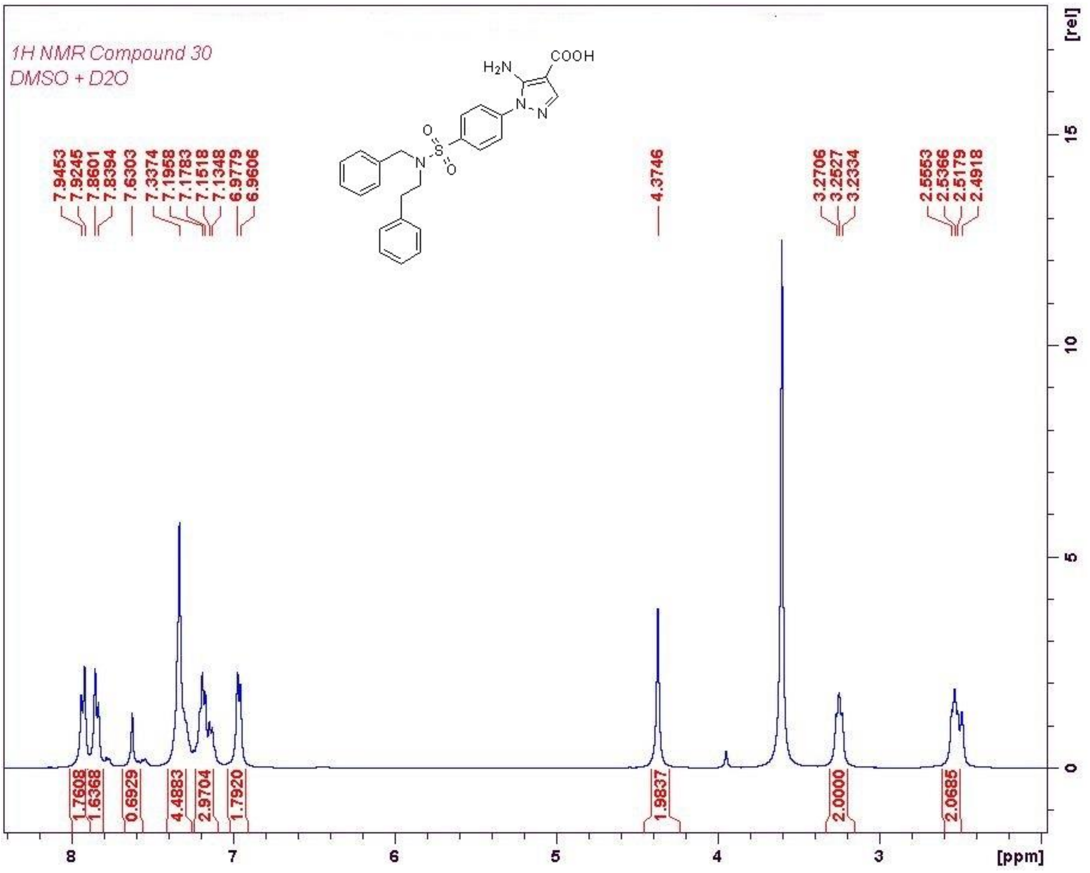

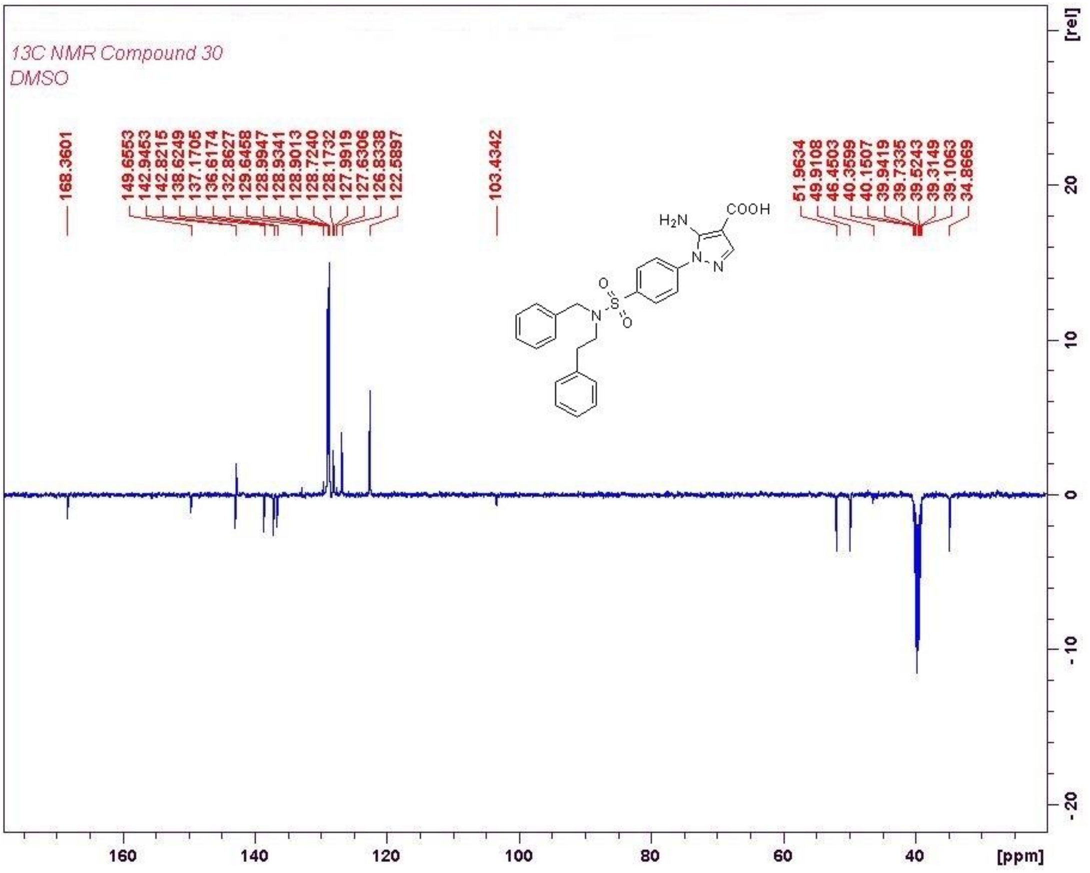

Table S1. Elemental analysis

Comp.	Formula (MW)	Anal. Calcd.				Anal. Found		
		C	H	N		C	H	N
**3a**	$C_{17}H_{15}NO_3S$ (313.37)	65.16	4.82	4.47		65.42	4.84	4.49
**3b**	$C_{18}H_{17}NO_3S$ (327.40)	66.04	5.23	4.28		66.30	5.25	4.30
**3c**	$C_{19}H_{19}NO_3S$ (341.43)	66.84	5.61	4.10		66.57	5.59	4.08
**3d**	$C_{20}H_{21}NO_3S$ (355.45)	67.58	5.96	3.94		67.30	5.94	3.92
**3e**	$C_{21}H_{23}NO_3S$ (369.48)	68.27	6.27	3.79		68.54	6.29	3.80
**4**	$C_{18}H_{15}NO_5S$ (357.38)	60.50	4.23	3.92		60.74	4.25	3.93
**5**	$C_{18}H_{17}NO_3S$ (327.40)	66.04	5.23	4.28		66.30	5.25	4.30
**7a**	$C_{21}H_{22}N_2O_6S_2$ (462.54)	54.53	4.79	6.06		54.31	4.77	6.03
**7b**	$C_{23}H_{26}N_2O_6S_2$ (490.59)	56.31	5.34	5.71		56.08	5.32	5.69
**7c**	$C_{25}H_{30}N_2O_6S_2$ (518.64)	57.90	5.83	5.40		57.67	5.80	5.38
**7d**	$C_{31}H_{26}N_2O_6S_2$ (586.68)	63.47	4.47	4.78		63.72	4.49	4.80
**7e**	$C_{33}H_{30}N_2O_6S_2$ (614.73)	64.48	4.92	4.56		64.22	4.90	4.54
**7f**	$C_{32}H_{28}N_2O_6S_2$ (600.70)	63.98	4.70	4.66		63.72	4.68	4.64
**7g**	$C_{25}H_{22}N_2O_6S_2$ (510.58)	58.81	4.34	5.49		58.57	4.32	5.47
**8**	$C_{17}H_{13}NO_4S$ (327.35)	62.37	4.00	4.28		62.62	4.01	4.30
**9**	$C_{25}H_{29}N_2NaO_6S_2$ (540.62)	55.54	5.41	5.18		55.76	5.43	5.20
**13**	$C_{23}H_{19}NO_8S$ (469.46)	58.84	4.08	2.98		59.07	4.10	2.99
**17**	$C_{10}H_{10}N_4O_4S$ (282.27)	42.55	3.57	19.85		42.72	3.58	19.92
**18a**	$C_{12}H_{14}N_4O_4S$ (310.33)	46.45	4.55	18.05		46.36	4.53	17.98
**18b**	$C_{13}H_{16}N_4O_4S$ (324.36)	48.14	4.97	17.27		48.33	4.99	17.33
**18c**	$C_{14}H_{18}N_4O_4S$ (338.38)	49.69	5.36	16.56		49.49	5.34	16.49
**19a**	$C_{14}H_{18}N_4O_4S$ (338.38)	49.69	5.36	16.56		49.49	5.34	16.49
**19b**	$C_{16}H_{22}N_4O_4S$(366.44)	52.44	6.05	15.29		59.28	3.97	5.53
**19c**	$C_{19}H_{28}N_4O_4S$ (408.52)	55.86	6.91	13.71		55.64	6.88	16.65
**23**	$C_{17}H_{16}N_4O_4S$ (372.40)	54.83	4.33	15.05		54.61	4.31	14.99
**24**	$C_{24}H_{22}N_4O_4S$ (462.52)	62.32	4.79	12.11		62.56	4.80	12.16
**28**	$C_{18}H_{18}N_4O_4S$ (386.43)	55.95	4.70	14.50		55.72	4.68	14.44
**29**	$C_{26}H_{26}N_4O_4S$ (490.58)	63.66	5.34	11.42		63.91	5.36	11.46
**30**	$C_{25}H_{24}N_4O_4S$ (476.55)	63.01	5.08	11.76		63.26	5.10	11.80

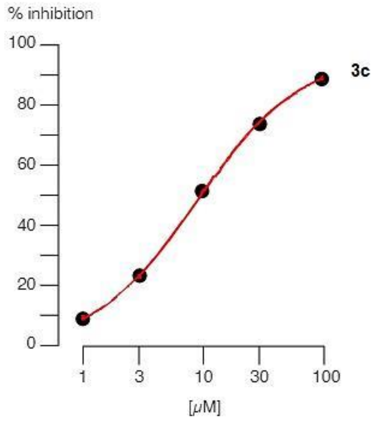

Figure S1. Quantitative analysis of the % inhibition of Panx1-mediated membrane currents in oocytes for compound **3c**.

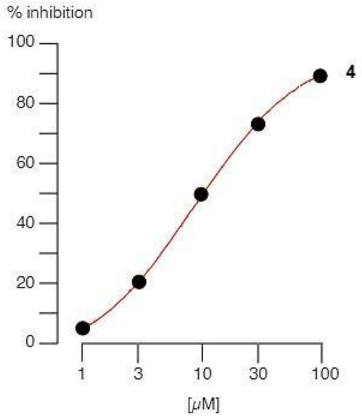

Figure S2. Quantitative analysis of the % inhibition of Panx1-mediated membrane currents in oocytes for compound **4**.

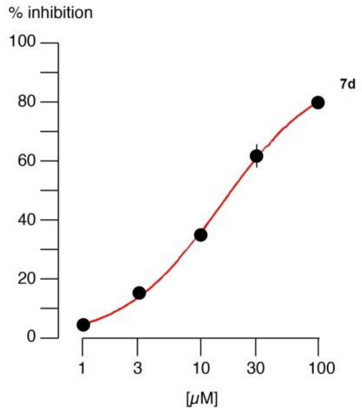

Figure S3. Quantitative analysis of the % inhibition of Panx1-mediated membrane currents in oocytes for compound **7d**.
